# Surface‐Embedding of Mo Microparticles for Robust and Conductive Biodegradable Fiber Electrodes: Toward 1D Flexible Transient Electronics

**DOI:** 10.1002/advs.202206186

**Published:** 2023-03-30

**Authors:** Jinho Kim, Congqi Yang, Taehyun Yun, Seohyun Woo, Hwajoong Kim, Mugeun Lee, Minji Jeong, Hyeji Ryu, Namjung Kim, Seongjun Park, Jaehong Lee

**Affiliations:** ^1^ Department of Robotics and Mechatronics Engineering DGIST 333, Techno jungang‐daero, Hyeonpung‐eup, Dalseong‐gun Daegu 42988 Republic of Korea; ^2^ Department of Bio and Brain Engineering Korea Advanced Institute of Science and Technology (KAIST) 291 Daehak‐ro, Yuseong‐gu Daejeon 34141 Republic of Korea; ^3^ Department of Mechanical Engineering Gachon University 1342, Seongnam‐daero, Sujeong‐gu, Seongnam‐si Gyeonggi‐do 13120 Republic of Korea

**Keywords:** biodegradable electronics, fiber electrode, flexible electronics, implantable electronics

## Abstract

Fiber‐based implantable electronics are one of promising candidates for in vivo biomedical applications thanks to their unique structural advantages. However, development of fiber‐based implantable electronic devices with biodegradable capability remains a challenge due to the lack of biodegradable fiber electrodes with high electrical and mechanical properties. Here, a biocompatible and biodegradable fiber electrode which simultaneously exhibits high electrical conductivity and mechanical robustness is presented. The fiber electrode is fabricated through a facile approach that incorporates a large amount of Mo microparticles into outermost volume of a biodegradable polycaprolactone (PCL) fiber scaffold in a concentrated manner. The biodegradable fiber electrode simultaneously exhibits a remarkable electrical performance (≈43.5 Ω cm^−1^), mechanical robustness, bending stability, and durability for more than 4000 bending cycles based on the Mo/PCL conductive layer and intact PCL core in the fiber electrode. The electrical behavior of the biodegradable fiber electrode under the bending deformation is analyzed by an analytical prediction and a numerical simulation. In addition, the biocompatible properties and degradation behavior of the fiber electrode are systematically investigated. The potential of biodegradable fiber electrode is demonstrated in various applications such as an interconnect, a suturable temperature sensor, and an in vivo electrical stimulator.

## Graphical abstract

A biodegradable fiber electrode with high electrical conductivity and strong mechanical robustness is fabricated using a surface‐embedding of Mo microparticles into a biodegradable fiber scaffold. The fiber electrode shows excellent biocompatibility and dissolution properties against biofluids. This study also provides several demonstrations of the fiber electrode such as an interconnect, suturable fiber temperature sensor, and in vivo electrical stimulator.

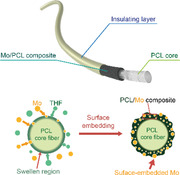

## 1 Introduction

Implantable sensing devices, which can be directly inserted into the human body, bear promise in improving healthcare^[^ ^1^, ^2^, ^3^ ^]^ and rehabilitation^[^ ^4^, ^5^, ^6^ ^]^ by continuously providing biological information such as electrocorticography,^[^ ^7^, ^8^ ^]^ electrocardiography,^[^ ^9^, ^10^ ^]^ electromyography,^[^ ^11^, ^12^ ^]^ inflammation,^[^ ^13^, ^14^ ^]^ and biomechanical signals.^[^ ^15^, ^16^ ^]^ In this regard, several implantable devices based on soft materials have been intensively developed due to high mechanical compliance and adaptability which prevents early device failure and severe inflammatory response.^[^ ^17^, ^18^ ^]^ However, a few practical issues of soft implantable devices have still limited their usage in clinical practice. For instance, the structural mismatch between existing planar devices and most organs with complex structures could induce nonconformal interfaces and practical difficulties in applying the devices to target organs.^[^ ^19^, ^20^ ^]^ In addition, the solid fixation of soft devices onto moving and pulsatile organs in the body is practically challenging in clinical situations.^[^ ^21^, ^22^ ^]^ To overcome the practical limitations of previous 2D implantable devices, fiber‐based implantable devices, which can also act as a medical suture at the same time, have been recently developed.^[^ ^23^, ^24^ ^]^ Our group developed a wireless and suturable fiber strain‐sensing system that can be directly sutured onto target tissues for the continuous monitoring of physiological strains of connective tissues.^[^ ^25^ ^]^ Kalidasan et al. also developed a wireless sensing suture that can measure physicochemical states in deep surgical sites.^[^ ^26^ ^]^ These fiber‐based implantable sensing devices not only successfully overcame the structural limitations of previous implantable devices but also provide intimate integration and stable fixation of the devices with target organs in surgical procedures. Nevertheless, existing fiber‐based implantable devices still require surgical extraction of the devices after use, which makes the devices difficult to be applied to clinical practice.

Recent developments of biodegradable devices, which can be dissolved by biofluids, provide great potential to avoid the need for a second surgical procedure to extract implanted devices after use.^[^ ^27^, ^28^, ^29^, ^30^, ^31^ ^]^ However, it has been challenging to develop fiber‐based biodegradable devices yet because it is difficult to fabricate biodegradable fiber electrodes that are not only fully composed of biodegradable materials in the form of fiber but also achieve high electrical and mechanical properties simultaneously. Hu et al. developed a cellulose‐based biodegradable and conductive microfibers using bacterial cellulose hydrogel incorporated with carbon nanotubes and polypyrrole.^[^ ^32^ ^]^ Their microfibers exhibited high tensile strength based on the wet‐stretching and twisting process of the fiber; However, despite the use of nonbiodegradable conducting materials such as carbon nanotubes and polypyrrole, the electrical conductivity of the microfiber was limited compared to that of metal‐based electrode. Such limited electrical conductivity could constraint their applicability to wireless readout systems, which are essential for implantable applications. Despite the high demand in implantable applications, biodegradable fiber electrodes with high electrical and mechanical performance, to the best of our knowledge, have been barely developed yet.

In this article, we present a facile approach for fabricating a biodegradable fiber electrode which simultaneously exhibits high electrical conductivity and mechanical robustness. The fiber electrode is fabricated by concentrating a large amount of Mo microparticles in the outermost volume of a polymeric fiber scaffold (surface‐embedding process). The fabricated biodegradable fiber electrode exhibits a considerably low electrical resistance (≈43.5 Ω cm^−1^) based on the densely embedded Mo microparticles in the conductive layer of the fiber electrode. In addition, although the huge amount of Mo microparticles are embedded into the polymeric fiber scaffold, the fiber electrode maintained excellent mechanical robustness and high stability for ≈4000 bending cycles, thanks to an intact polymeric core in the fiber electrode. The analytical and numerical model are developed to explain the electrical behavior of the fiber electrode under bending deformation. The biocompatibility and dissolution property of the biodegradable fiber electrode are investigated for its usage in practical applications. We demonstrate the biodegradable fiber electrode in various applications such as a stable interconnect for electrical circuits, a suturable temperature sensor, and an in vivo electrical stimulator in a rat model.

## 2 Result and Discussion

### 2.1 Fabrication and Characterizations of the Polycaprolactone Scaffold Fiber

**Figure 1:**
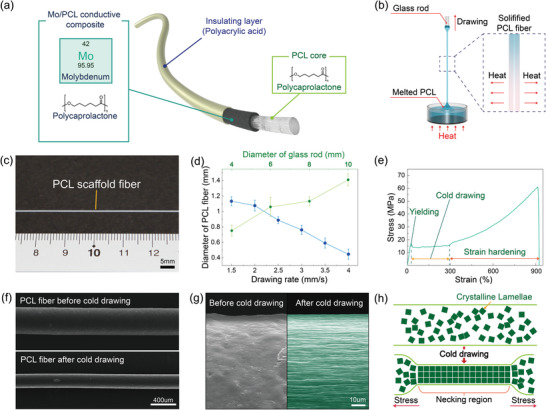
a) Schematic illustration of the biodegradable fiber electrode based on the PCL core and the conductive composite layer between PCL and Mo microparticles. b) Schematic illustration of the melt drawing process for fabricating the PCL scaffold fiber. c) Photograph of the PCL scaffold fiber fabricated by the melt drawing process. d) Diameter change of the drawn PCL scaffold fibers according to various drawing rates (blue line) and different diameters of the glass rod (green line). Data are presented as mean ± SD (*n* = 3) e) Stress–strain curve of the PCL scaffold fiber, showing yielding, cold drawing, and strain hardening behavior of the fiber. f) SEM images of the PCL scaffold fiber (upper) and the cold‐drawn PCL fiber (lower), showing a decrease in the diameter of the fiber. g) SEM images of the PCL scaffold fiber (left) and the cold‐drawn PCL fiber(right) with the rearrangement of crystalline structures. h) Schematic illustration describing the rearrangement of crystalline lamellae in the PCL fiber after the cold drawing process.

We developed a high‐performance biodegradable fiber electrode using molybdenum (Mo) microparticles and a biodegradable polymer, polycaprolactone (PCL). **Figure** **1**a presents a schematic illustration of the biodegradable fiber electrode consisting of a Mo/PCL conductive composite layer and PCL core fiber. According to the desired applications, an insulating layer, such as polyacrylic acid (PAA), poly(lactide‐co‐glycolide) (PLGA), poly(l‐lactic acid) (PLLA), polyvinyl alcohol (PVA), can also be coated onto the fiber electrodes to ensure no electrical short with the surroundings. The fabrication process of biodegradable fiber electrodes involves two main steps: melt‐drawing of the PCL scaffold fiber^[^ ^33^ ^]^ and surface‐embedding of Mo microparticles for the formation of the conductive composite layer in the scaffold fiber. Figure 1b shows a schematic illustration of the melt‐drawing process for the PCL scaffold fiber. The PCL fiber was efficiently drawn by a glass rod from the melted PCL liquid, followed by solidification of the drawn fiber through cooling with ambient air. Through the melt‐drawing process, a PCL scaffold fiber with a cylindrical structure was successfully obtained with high uniformity and scalability up to tens of meters (Figure 1c, Figures S1a,b, Supporting Information). The cross‐sectional structure of the PCL scaffold fiber could also be adjusted to various shapes including rectangular, triangular, and oval structure by changing the shape of the rod accordingly (Figure S2a, Supporting Information). The diameter of the drawn PCL scaffold fiber could be controlled by adjusting the drawing speed, diameter of rod, and melting temperature. The increase in the drawing rate results in the decrease in a diameter of the drawn PCL fiber because fast drawing leads to more elongational flow of the drawn PCL liquid to allow thin semi‐hyperbolic shapes before its solidification (Figure 1d). In a similar manner, high temperature for the melted PCL liquid decreased the diameter of the drawn PCL scaffold fiber (Figure S2b, Supporting Information). The PCL liquid melted by the higher temperature requires a longer time to be solidified during the drawing process, resulting in more semi‐hyperbolic deformation of the drawn PCL liquid and a reduced diameter of the PCL scaffold fiber. In addition, the diameter of the drawn PCL fiber could also be adjusted by changing the diameter of the glass rod. An increase in the diameter of the rod could draw more melted PCL liquid, producing the PCL fiber with increased diameter (Figure 1d).

Figure 1e shows the stress–strain behavior of the PCL scaffold fiber during uniaxial tensile stretching. The PCL scaffold fiber exhibited the typical mechanical property of semicrystalline polymers, which includes elastic, yielding, necking (cold drawing), and strain‐hardening behavior.^[^ ^34^, ^35^ ^]^ In this study, the PCL scaffold fiber produced by the melt‐drawing process was consecutively cold drawn at a strain of ≈320%, which induced plastic deformation of the fiber, to improve its mechanical property (Figure S3a, Supporting Information). In addition to the elongation of ≈320% in the fiber, the diameter of the cold‐drawn PCL fiber was dramatically decreased to ≈240 µm, which is about 2‐fold thinner than that of the original PCL scaffold fiber without cold‐drawing (Figure 1f). The diameter of the cold‐drawn PCL fiber can be further decreased by decreasing the diameter of the PCL scaffold fiber during the melt‐drawing process. The diameter of the PCL scaffold fiber was additionally decreased to ≈260 µm by using a smaller glass rod with 2 mm diameter in the melt‐drawing process (Figure S4a, Supporting Information). In addition, the diameter of the PCL scaffold fiber was further decreased to ≈145 µm through the cold‐drawing process (Figure S4b, Supporting Information). The significant change in the length and diameter of the cold‐drawn PCL fiber is attributed to the large‐scale morphological rearrangement of the stacked crystalline lamellae in the semicrystalline polymeric fiber (Figure 1h).^[^ ^36^, ^37^ ^]^ According to the rearrangement of the crystalline structure, an aligned fibril structure along the longitudinal direction was observed on the surface of the cold‐drawn PCL fiber (Figure 1g). Based on its highly oriented fibrillar crystalline structure, the cold‐drawn PCL fiber exhibited higher mechanical strength than the PCL fiber without cold‐drawing, providing improved mechanical property (Figure S3b, Supporting Information). In addition, the decreased diameter of the cold‐drawn PCL fiber leads to decrease in flexural stiffness of the fiber according to the following equation^[^ ^38^, ^39^ ^]^

(1) $$D=\frac{Eh^{3}}{12\left(1-v^{2}\right)}$$

where *D* is the flexural stiffness, *E* means the elastic modulus, *h* indicates the thickness of the materials, and *v* is Poisson's ratio of materials. The calculated flexural stiffness of the cold‐drawn PCL fiber (0.208 N mm) has 3.2 times lower than that of the PCL fiber without cold‐drawing (0.664 N mm), demonstrating high compliance of the cold‐drawn PCL fiber. The low flexural stiffness of the cold‐drawn fiber is highly beneficial for minimizing foreign body reaction against external materials in in vivo applications.^[^ ^39^ ^]^

### 2.2 Surface‐Embedding of Mo Microparticles on Polycaprolactone (PCL) Fiber

**Figure 2:**
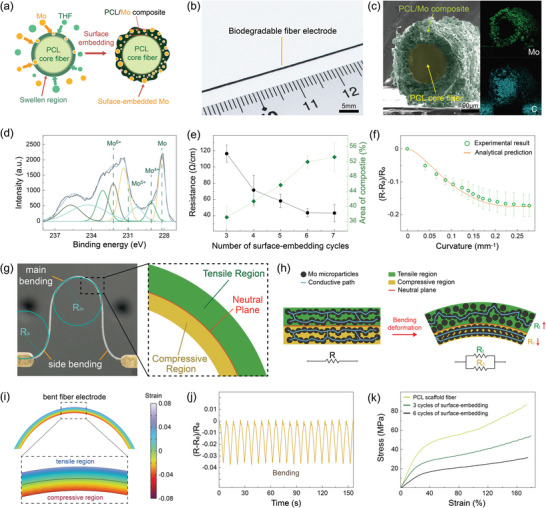
a) Schematic illustration of the surface‐embedding process, showing the embedding of Mo microparticles into the soft PCL region dissolved by tetrahydrofuran. b) Photograph of the fabricated biodegradable fiber electrode. c) Cross‐sectional SEM image and the corresponding EDS mapping images of the biodegradable fiber electrode, indicating the distribution of Mo microparticles in a conductive composite layer of the fiber electrode. d) Atomic bonding energy of the surface of the biodegradable fiber electrode analyzed by XPS. e) Electrical resistance (black line) per unit length of the biodegradable fiber electrode and cross‐sectional areal portion of the conductive composite layer (green line) according to the number of repeated surface‐embedding processes. Data are presented as mean ± SD (*n* = 3). f) Resistive response of the biodegradable fiber electrode under bending deformation. The solid line indicates the calculated expectation of resistive response under bending deformation based on an analytical formula. Data are presented as mean ± SD (*n* = 3). g) Photograph and schematic illustration showing the biodegradable fiber electrode under the bending deformation. h) Schematic illustration of the change of the electrical pathway for the biodegradable fiber electrode divided into tensile and compressive regions under bending deformation. i) Numerical simulation of the strain distribution on bent fiber electrode in the main bending. j) Stability test of the biodegradable fiber electrode against repeated bending deformation with curvature from 0.04 mm^−1^ to 0.09 mm^−1^. k) Stress–strain curves of the biodegradable fiber electrodes fabricated with different number of the repeated surface‐embedding process (0, 3, 6 cycles).

For achieving high electrical conductivity, a large amount of Mo microparticles were effectively embedded into outermost volume of the cold‐drawn PCL fiber via a surface‐embedding process. **Figure** **2**a shows a schematic illustration of the surface‐embedding process of the Mo microparticles for fabricating a biodegradable fiber electrode. For the surface‐embedding of the Mo microparticles, the cold‐drawn PCL fiber was vigorously reacted with a solution of Mo microparticles in a mixture of tetrahydrofuran and tetraethylene glycol by physically shaking the system. During the process, the surface of the cold‐drawn PCL fiber is swollen by tetrahydrofuran molecules diffused into the polymeric matrix, enabling the efficient embedding of Mo microparticles in the swollen region of the fiber. Figure 2b presents photograph of the fabricated biodegradable fiber electrode, showing the change in the color of the fiber due to the Mo microparticles embedded in the PCL scaffold fiber. Cross‐sectional scanning electron microscopy (SEM) image of the fiber electrode with a diameter of ≈307 µm reveals that the electrode consists of an intact PCL scaffold fiber at the core and a conductive composite layer between the PCL fiber and Mo microparticles along the outer edge of the electrode (Figure 2c). The uniform and dense distribution of Mo microparticles with an average diameter of ≈2.54 µm over the composite region of the fiber electrode was clearly identified in the Energy Dispersive X‐ray Spectroscopy (EDS) image of the cross‐section of the electrode (Figure 2c and Figure S5, Supporting Information). The successful embedding of the Mo microparticles in the fiber electrode was also investigated by measuring atomic bonding energy by X‐ray photoelectron spectroscopy (XPS) of the electrode (Figure 2d). The metallic Mo peak (228.1 eV) and several peaks of Mo oxides (229.0, 230.3, and 232.2 eV for Mo^4+^, Mo^5+^, and Mo^6+^, respectively) in the XPS graph present the presence of the Mo microparticles in the fiber electrode.

The densely embedded Mo microparticles in the fiber electrode provide excellent conductive pathways via the contacts between the embedded Mo microparticles, resulting in high electrical performance of the fiber electrode. Figure 2e shows the electrical resistance per unit length (Ω cm^−1^) of the presented biodegradable fiber electrode. The electrical resistance of the fiber electrode substantially decreased to ≈43.5 Ω cm^−1^, corresponded to ≈2252.6 S m^−1^, by repeating the surface‐embedding process of Mo microparticles. The enhancement of the electrical conductivity of the fiber electrode upon repetitive surface‐embedding process is mainly attributed to a considerable increase in the amount of Mo microparticles in the fiber that results in further dense connections between the Mo microparticles (Figure S6a, Supporting Information). In addition, the cross‐sectional area of the conductive composite in the fiber electrode gradually increased as the surface‐embedding process is repeated, contributing to a decrease in the electrical resistance of the fiber electrode (Figure 2e). The electrical conductivity of the conductive composites in the fiber electrode can be theoretically explained by the 3D percolation theory and classical power‐law relationship

(2) $$\sigma =\sigma_{0}{\left(V_{f}-V_{p}\right)}^{s}$$

where *σ* is the electrical conductivity of the biodegradable fiber electrode, *σ*_0_ is the bulk conductivity of Mo, *V_f_*, and *V_p_* indicate the volume fraction of Mo microparticles in the conductive composite layer of the fiber electrode and percolation threshold, respectively, and *s* is a critical exponent.^[^ ^40^ ^]^ A detailed explanation regarding the calculated expectation of the electrical conductivity of the fiber electrodes based on 3D percolation theory is given in the Supporting Information. Figure S6b (Supporting Information) shows the electrical resistance of the fiber electrode according to the volume fraction of Mo microparticles in the conductive composite layer of the fiber electrode. The change in the electrical resistance of the fiber electrode showed excellent agreement with the calculated expectation derived from 3D percolation theory.

The electrical performance of the biodegradable fiber electrode can also be optimized by adjusting the reaction time for the surface‐embedding process, concentration of the Mo microparticles and tetraethylene glycol used in the fabrication process. Figure S7a,b (Supporting Information) exhibit the electrical resistance of the biodegradable fiber electrode according to the reaction time and concentration of the Mo microparticles for the surface‐embedding process, respectively. The electrical resistance of the fiber electrode clearly decreased with increasing reaction time and concentration of Mo microparticles due to the increased amount of Mo microparticles embedded in the conductive composite layer of the fiber electrode. The PCL core in the fiber electrode might also be damaged if the reaction time for the surface‐embedding process is excessively long. However, the surface‐embedding process can be effectively optimized without damaging the PCL core by adjusting the reaction time. In this work, the reaction time of 5s was used for the optimized surface‐embedding process of Mo microparticles in which the PCL core is not damaged. The electrical resistance of the fiber electrode was not further decreased with respect to longer reaction time than the optimized condition (5 s) due to the potential damage of the PCL fiber. Instead, the electrical property of the fiber electrode could be improved using the repeated surface‐embedding process which results in the larger amount of Mo microparticles embedded in the fiber electrode. The addition of tetraethylene glycol can also enhance the electrical performance of the fiber electrode by improving the uniform dispersion of Mo microparticles and reducing Mo microparticle agglomeration in the electrode.^[^ ^41^, ^42^, ^43^ ^]^ Figure S7c (Supporting Information) shows the electrical resistance of the fiber electrode with respect to the increasing concentration of tetraethylene glycol used in the surface‐embedding process, demonstrating the improvement of the electrical performance of the fiber electrode thanks to the use of the tetraethylene glycol. The Mo microparticles embedded in the fiber electrode were intactly maintained without any considerable delamination even under bending deformation of the fiber electrode with various curvatures (Figure S8, Supporting Information). This shows high stability and good adhesion between the embedded Mo microparticles and PCL fiber matrix based on van der Waals bond between the two components.^[^ ^44^, ^45^ ^]^

Figure 2f displays the resistive response of the fiber electrode under its bending deformation with various curvatures. Interestingly, the electrical resistance of the fiber electrode decreased with increasing curvature during the bending deformation. This unusual behavior of the fiber electrode can be explained by the improved percolation of Mo microparticles in the compressive region of the bent electrode. In particular, the bending deformation of the fiber electrode leads to the development of two regions in the electrode: compressive and tensile regions (Figure 2g). The two regions have different volume changes upon bending deformation, resulting in the change of the percolated network of Mo microparticles in each region (Figure 2h,i). The percolated network of Mo microparticles in the compressive region of the electrode becomes denser upon bending deformation, and vice versa in the tensile region. These changes in the percolated network of the Mo microparticles in each region also result in a change in the electrical conductivity of each region according to the 3D percolation theory. Therefore, the fiber electrode can be described as two different resistors connected in parallel under bending deformation (Figure 2h). Based on the simple equivalent circuit of the fiber electrode and 3D percolation theory, the total electrical resistance of the fiber electrode under bending deformation can be calculated as follows

(3) $$\begin{array}{ccc} & & R_{\mathrm{total}}=\frac{1}{\sigma_{0}}\left[\frac{L_{b1}}{{\left(\frac{V_{Mt1}^{0}}{V_{t1}}-V_{p}\right)}^{s}A_{t1}+{\left(\frac{V_{Mc1}^{0}}{V_{c1}}-V_{p}\right)}^{s}A_{c1}}\right.\\ & & \;\left.+\;\frac{2L_{b2}}{{\left(\frac{V_{Mt2}^{0}}{V_{t2}}-V_{p}\right)}^{s}A_{t2}+{\left(\frac{V_{Mc2}^{0}}{V_{c2}}-V_{p}\right)}^{s}A_{c2}}+\frac{2L_{n}}{{\left(V_{f}-V_{p}\right)}^{s}A_{n}}\right] \end{array}$$

where *σ*_0_ is the electrical conductivity of Mo, *L*_*b*1_ and *L*_*b*2_ mean the lengths of the bent electrode at the main and side bending, respectively, *V_t_* and *V_c_* indicate the volume of tensile and compressive region of the bent electrode, respectively, *A_t_* and *A_c_* are the cross‐sectional areas of the tensile and compressive regions of the bent electrode, $V_{Mt}^{0}$ and $V_{Mc}^{0}$ are the volume of Mo microparticles before bending deformation in the tensile and compressive regions of the bent electrode, respectively, *V_p_* is the percolation threshold, *V_f_* is the volume fraction of Mo microparticles in the unbent state, s is a critical exponent, and *L_n_* and *A_n_* are the length and cross‐sectional area of unbent fiber electrode, respectively. Detailed calculations of the electrical resistance of the fiber electrode under bending deformation are described in the Supporting Information. The calculated electrical resistance of the fiber electrode according to the bending deformation closely matched with the experimental results as shown in Figure 2f. Although the electrical resistance of the fiber electrode was slightly changed against the mechanical deformation, it was stably recovered after the bending deformation was removed, demonstrating the high stability of the fiber electrode (Figure 2j). The stable resistive response of the fiber electrode was retained without considerable degradation even after a 4000‐cyclic bending test with the curvature from 0.04 to 0.09 mm^−1^, showing the excellent durability of the electrode (Figure S15a, Supporting Information). In addition, the electrical resistance of the fiber electrode was also stable for several weeks in air condition, demonstrating the excellent long‐term stability of the fiber electrode against atmospheric condition (Figure S15b, Supporting Information). This high durability of the biodegradable fiber electrode is highly advantageous compared with typical bulk transient metal wires such as a Mo wire. Due to the rigidity and fatigue properties, the bulk Mo wire is mechanically vulnerable to repeated bending deformation, showing poor durability (Figure S16, Supporting Information).

The mechanical behavior of the fiber electrode was also evaluated as shown in Figure 2k. Although typical conductive composites with high electrical conductivity are easy to experience severe degradation of mechanical strength by conductive fillers,^[^ ^46^, ^47^, ^48^ ^]^ the presented fiber electrode exhibited high mechanical strength despite the large amount of Mo microparticles in the electrode (Figure 2k). The mechanical strength of the fiber electrode was lower than that of the cold‐drawn PCL fiber owing to the embedded Mo microparticles, but the fiber electrode showed stable and comparable mechanical properties due to the intact PCL core in the electrode. This is highly advantageous compared to previously reported conductive composite‐based fiber electrodes, which are typically difficult to simultaneously achieve high electrical and mechanical properties.

### 2.3 Biocompatible and Dissolution Properties of Biodegradable Fiber Electrodes

**Figure 3:**
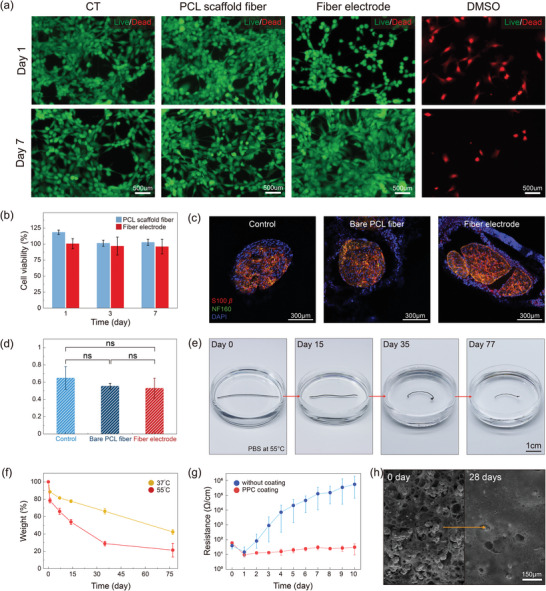
a) Fluorescence images of live (green)/dead (red) 3T3 fibroblasts cells cultured with a petri dish (CT), the cold‐drawn PCL fiber, the biodegradable fiber electrode, and DMSO for 7 days. b) Cell viability on the cold‐drawn PCL fiber and biodegradable fiber electrode measured using the CCK‐8 assay for 7 days. Data are presented as mean ± SD (*n* = 3). c) The IHC staining images of mice sciatic nerve for NFM (neurofilament) and S100 (Schwann cell) (blue: DAPI, red: S100, green: NFM) after 6‐day implantation of the control, bare PCL fiber, and the biodegradable fiber electrode. DAPI was used as nucleic acid marker. d) Quantification of the percentage of cells that are S‐100 positive in sciatic nerve from mice in control, bare PCL fiber and fiber electrode group. Data are presented as mean ± SD (*n* = 3), and statistical analysis proceeded with one‐way ANOVA (*P* > 0.05; ns: no significant difference). e) Photographs of the biodegradable fiber electrode at various states of dissolution in PBS (pH 7.4, 55 °C). f) Weight loss of the biodegradable fiber electrode in PBS (pH 7.4, 37°C (yellow), and 55 °C (red)). Data are presented as mean ± SD (*n* = 3). g) Change in the electrical resistance of the biodegradable fiber electrode with and without the PPC buffer coating layer in PBS (pH 7.4, 37 °C) over time. Data are presented as mean ± SD (*n* = 3). h) SEM images of the biodegradable fiber electrode in PBS (pH 7.4, 37 °C) at 0 and 28 days, showing the change of the surface morphologies of the biodegradable fiber electrode.

To evaluate the biocompatibility of the biodegradable fiber electrodes, a live/dead test was performed using NIH/3T3 fibroblasts cells. We cultured 3T3 fibroblast cells with a typical Petri dish, cold‐drawn PCL fiber, biodegradable fiber electrode, and Dimethyl sulfoxide (DMSO). **Figure** **3**a shows no significant difference in cell viability among the control group, the bare PCL fiber group as well as the coated PCL fiber group. The similar results are observed when fibers were incubated in cells for 1 day or 7 days. In addition, a cell counting kit‐8 (CCK‐8) assay was also conducted with NIH/3T3 fibroblasts cells to quantify the cell viability of the biodegradable fiber electrodes. As shown in Figure 3b, even after 7 days of incubation of fiber electrodes in cells, the cell viability is observed to be over 90%, demonstrating the excellent biocompatibility of the fiber electrodes (Figure 3b). Although Mo is one of biodegradable and biocompatible materials, excessive intake of Mo can be toxic for humans. However, the dissolution rate of Mo microparticles in the fiber electrode (5.28 µg cm^−1^ day^−1^) is significantly smaller than a tolerable upper intake level of Mo (2 mg day^−1^) recommended by the Food and Nutrition Board,^[^ ^49^ ^]^ showing the safety of the fiber electrode in in vivo applications. In addition, an in vivo biocompatibility of the biodegradable fiber electrode was investigated. To this end, we implanted the biodegradable fiber electrodes and cold‐drawn PCL fibers (*n* = 3) on the sciatic nerve of a rat model, and performed immunohistochemistry (IHC) staining of Iba1, S100*β*, and NF160 on the sciatic nerve after 6 days. Figure 3c and Figures S17 and S18 (Supporting Information) show the IHC staining images of the mice's sciatic nerve 6 days after the implantation of the control group (bare), cold‐drawn PCL fiber, and fiber electrode. The S100*β* and NF160 were densely distributed over the sciatic nerve after 6 days of the implantation, demonstrating the excellent in vivo biocompatibility of the fiber electrode (Figure 3c). The amount of the S100*β* positive cells in sciatic nerve with the fiber electrode was comparable with that of the control group, meaning that the fiber electrode has no cytotoxicity and excellent biocompatibility (Figure 3d). In addition, the Iba1 on the sciatic nerve with the fiber electrode was comparable with that of the control group, showing no remarkable inflammation on the sciatic nerve (Figure S18, Supporting Information). The fluorescence intensity of the Iba1 on the sciatic nerve with the fiber electrode was comparable with that of the control group, showing no remarkable inflammation on the sciatic nerve (Figure S19, Supporting Information). Future work about the in vivo biocompatibility test is, however, required to demonstrate chronic in vivo use of the fiber electrode. To this end, the in vivo biocompatibility and immunohistology analysis of the fiber electrode should be performed for longer time, at least 12 weeks, to investigate chronic in vivo biocompatibility of the fiber electrode.

To investigate the biodegradable performance of the presented biodegradable fiber electrode, the fiber electrode was immersed in phosphate buffered saline (PBS), pH 7.4. Figure 3e shows the efficient dissolution of biodegradable fiber electrodes in the buffer solution (55 °C) over time. After 77 days, the biodegradable fiber electrode was effectively dissolved in PBS solution. In particular, the weight of the biodegradable fiber electrode decreased to 42% after 77 days of the immersion in the PBS solution at 37 °C (Figure 3f). The biodegradable fiber electrode immersed in the PBS solution at 55 °C was dissolved ≈7% faster than that at 37 °C per week.

The electrical property of the biodegradable fiber electrode in the PBS solution was also investigated by measuring the electrical resistance of the fiber electrode in the PBS solution at 37 °C (Figure 3g). On the first day in the PBS solution, the electrical resistance of the fiber electrode dropped to ≈10 Ω cm^−1^ and then gradually increased over time. The initial decrease in the electrical resistance of the fiber electrode is mainly attributed to the disintegration of the nanoscale native‐oxide layers of the Mo microparticles, which obstruct the electrical pathway through the dense Mo microparticles.^[^ ^41^ ^]^ Consistent with the trend of the electrical resistance, the dense Mo microparticles on the surface of the conductive composite layer were dissolved after 28 days of immersion in the PBS solution at 37 °C (Figure 3h). The life time of the biodegradable fiber electrode can be effectively extended by coating a biodegradable buffer layer on the surface of the electrode for long‐term use of the electrode. The fiber electrode coated with polypropylene carbonate (PPC) successfully maintained its electrical resistance in the PBS solution over 10 days, thanks to the PPC‐based buffer layer (Figure 3g and Figure S20, Supporting Information). Although the Mo microparticles on the surface of the fiber electrode were dissolved in the PBS solution, the Mo microparticles embedded in the inner volume of the conductive composite layer remained until full degradation of the composite layer (Figure S21, Supporting Information). Because the dissolution rate of PCL is much slower than that of Mo, the full degradation time of the fiber electrode depends dominantly on the dissolution of PCL in the electrode. The full degradation time of the fiber electrode was calculated to ≈4 years by measuring the dissolution rate of the conductive composite layer and the PCL core in the fiber (Figure S22, Supporting Information). Moreover, the in vivo biodegradability of the fiber electrode was verified on a rat model. The biodegradable fiber electrode was implanted onto the sciatic nerve for 15 days (Figure S23a, Supporting Information). The weight of the biodegradable fiber electrode was decreased to 81.7% on the sciatic nerve of the rat after 15 days of the implantation (Figure S23b, Supporting Information), associated with the result of the in vitro biodegradation (Figure S22, Supporting Information). Similarly, most of the dense Mo microparticles on the surface of the fiber electrode were dissolved after 15 days of the implantation (Figure S23c, Supporting Information).

### 2.4 Demonstrations of the Biodegradable Fiber Electrode in Various Applications

**Figure 4:**
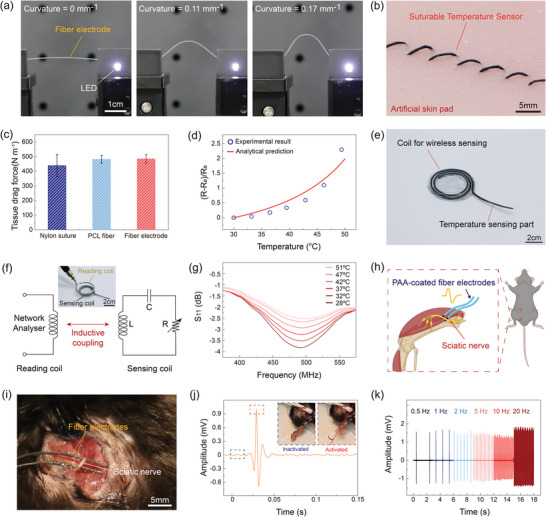
a) Photographs showing stable operation of a LED connected to the biodegradable fiber electrode under bending deformation. b) Photograph of the suturable fiber temperature sensor directly sutured on an artificial skin pad. c) Tissue drag force per unit circumference of the commercial nylon suture, cold‐drawn PCL fiber, and biodegradable fiber electrode, which is required to pull the sutures through the skin pad during the suturing process. Data are presented as mean ± SD (*n* = 3 trials). d) Resistive response of the suturable fiber temperature sensor under increasing temperature. The solid line shows the theoretical expectation of the resistive response of the sensor based on the analytical model. e) Photograph of a wireless temperature sensing system consisting of a single biodegradable fiber electrode. f) Schematic illustration of inductive coupling between the inductive coil in the temperature sensing system and an external reading coil from a network analyzer. g) The reflection coefficient (*S*_11_) spectra of the wireless temperature sensing system according to different temperatures from 28 °C to 51 °C, showing the change in the intensity of the *S*_11_ spectra. h) Schematic illustration showing electrical stimulation of the fiber electrodes on sciatic nerve of a rat model. i) Photograph of the PAA‐coated fiber electrodes applied to the sciatic nerve for electrical stimulation. j) The electrical signal generated by the electrical stimulation of the fiber electrodes on the sciatic nerve at 4 mA and 1 Hz. Inset images show the physical activation of the gastrocnemius muscle by the electrical stimulation. k) The electrical signals generated by the electrical stimulation of the fiber electrode with different frequencies at 0.5, 1, 2, 5, 10, and 20 Hz.

To demonstrate the electrical performance and stability of the biodegradable fiber electrode, the fiber electrode was used as an interconnect to turn on a light‐emitting diode (LED) as shown in **Figure** **4**a. The light intensity of the LED was stably maintained even under bending deformation of the biodegradable fiber electrode, showing the high electrical stability of the fiber electrode against mechanical deformation (Figure 4a). In addition, the biodegradable fiber electrode can be successfully used for suture‐type implantable electronic devices, one of promising electronic devices for biomedical applications.^[^ ^25^, ^26^, ^50^ ^]^ We chose to demonstrate a presented biodegradable fiber electrode as a suturable temperature sensor which can monitor the physiological temperature on target sites. Although a few noninvasive body temperature sensing methods in clinical practice, it is difficult for the existing sensing methods to precisely monitor local temperature of deep wound site in real time.^[^ ^51^ ^]^ Real‐time monitoring of the local temperature could be highly beneficial for detecting inflammatory responses during the wound healing process.^[^ ^52^ ^]^ For suturing of the biodegradable fiber electrode onto an artificial skin pad, a medical suturing thread was used as a guiding thread for the suturing process. The fiber temperature sensor directly connected to the suturing thread was sutured on the skin pad following the guiding thread sutured on the skin pad (Figure 4b and Figure S24, Supporting Information). To evaluate the mechanical stability of the fiber temperature sensor as a medical suture, the tissue drag force of the fiber temperature sensor was measured during the suturing process of the fiber sensor through the artificial skin (Figure 4c). The fiber temperature sensor and bare PCL fiber exhibited a tissue drag force of 484.67 and 481.76 N m^−1^, respectively, which was comparable with that of a medical‐grade nylon suture (438.98 N m^−1^), demonstrating the practically appropriate drag force of the fiber sensor that avoids tissue damage during suturing process.^[^ ^26^ ^]^ Figure 4d shows the relative change in electrical resistance of the suturable fiber temperature sensor upon applied temperature. The electrical resistance of the fiber temperature sensor was increased up to 2.5 times from the initial value against increasing temperature from room temperature to ≈50 °C. This exponential increase in the electrical resistance of the fiber sensor is attributed to the thermal expansion of the polymeric matrix of the fiber upon temperature.^[^ ^53^ ^]^ The thermal expansion of the fiber matrix under high temperature decreases the density of the percolated network of the Mo microparticles in the fiber sensor, resulting in the increase of the electrical resistance of the fiber sensor. According to the thermal expansion of the polymeric material and percolation theory related to the conductive composite, the resistive response of the fiber temperature sensor under varying temperature can be calculated as follows

(4) $$R_{\mathrm{temp}}=\frac{L_{0}}{\sigma_{0}A_{0}\left(1+\alpha \left(T_{1}-T_{0}\right)\right){\left(\frac{V_{Mo}}{V_{0}{\left(1+\alpha \left(T_{1}-T_{0}\right)\right)}^{3}}-V_{p}\right)}^{s}}$$

where *σ*_0_ is the bulk conductivity of Mo, *L*_0_ is the initial length of the fiber temperature sensor, *A*_0_ and *V*_0_ are the initial area and volume of the fiber sensor, respectively, *α* is the thermal expansion coefficient of PCL, *T*_0_ and *T*_1_ are the initial and increased temperatures, respectively, *V_Mo_* is the volume of Mo microparticles, *V_p_* is the percolation threshold, and s is a critical exponent. A detailed explanation of the calculated expectation is described in the Supporting Information. The calculated electrical resistance of the fiber temperature sensor under increasing temperature showed good agreement with the experimental data (Figure 4d), demonstrating that the presented biodegradable fiber electrode successfully can be used as a suturable temperature sensor. Furthermore, the fiber temperature sensor could maintain its stable response in the PBS solution by coating polypropylene carbonate (PPC) layer on the surface of the fiber sensor (Figure S26a, Supporting Information). Based on the encapsulation layer, the resistive response of the fiber temperature sensor was not affected by the PBS solution, providing the expected behavior of the sensor also in the PBS solution (Figure S26b, Supporting Information). In addition, the fiber temperature sensor can also be used to wirelessly monitor the temperature by forming a resistor–inductor–capacitor (RLC) circuit based on the biodegradable fiber electrode (Figure 4e and Figure S27, Supporting Information). The reflection coefficient *S*_11_ of the circuit was wirelessly monitored through the inductive coupling of the RLC sensor circuit with an external reading coil from a network analyzer (Figure 4f). The increase in the electrical resistance of the wireless sensing system induces the decrease in the intensity of the *S*_11_ spectra of the system, according to a decreased Q‐factor of a passive RCL circuit. The Q‐factor, related to the intensity and sharpness of the *S*_11_ spectra, is generally defined as follows

(5) $$Q=\frac{1}{R}\sqrt{\frac{L}{C}}$$

where *C*, *L*, and *R* indicate the capacitance, inductance, and electrical resistance of a passive wireless RLC circuit, respectively. Figure 4g shows the reflection coefficient (*S*_11_) spectra of the wireless fiber temperature sensing system according to different temperatures from 30 to 50 °C on the fiber sensor. The intensity of the *S*_11_ spectra of the wireless sensing system decreased with increasing temperature owing to the increase in the electrical resistance of the fiber temperature sensor in the system, which can be explained by Equation (5). To apply the wireless fiber temperature sensor in practical applications, however, full in vivo demonstration of the wireless sensing system should be further required.

In addition, the biodegradable fiber electrode was also evaluated as an in vivo electrical stimulator using a rat model as illustrated in Figure 4h. To prevent current leakage during electrical stimulation, poly (acrylic acid) (PAA) was coated on the surface of the fiber electrode as an insulating layer, partially exposing the tip (5 mm) of the fiber electrode for the electrical stimulation (Figures S28 and S29a, Supporting Information). The PAA is one of the widely used biodegradable polymers in several biomedical applications due to its nontoxicity, biodegradable property, high adhesion, and recyclability.^[^ ^54^, ^55^, ^56^, ^57^ ^]^ To study the effectiveness of fiber electrode as an electrical stimulator, stimulation experiment with fiber to the sciatic nerve was performed (Figure 4i). The PAA‐coated fiber electrodes were located between the sciatic nerve and underlying muscle tissue, maintaining contact between the exposed electrode and the sciatic nerve. Based on mucoadhesive property of the PAA layer, the fiber electrode was stably adhered to the muscle tissue during the in vivo demonstration.^[^ ^58^, ^59^ ^]^ To monitor the activity of stimulated muscle, EMG electrodes were inserted in a gastrocnemius muscle. Figure S29b (Supporting Information) displays the amplitude of the average EMG signals by controlling the current of electrical stimulation from 0.1 to 1.6 mA at 1 Hz using PAA coated fiber electrode. The activation of the gastrocnemius muscle was successfully observed according to modulating the amplitudes of electrical current without leakage of electrical current (Figure 4j and Video S1, Supporting Information). Moreover, the electrical stimulation was also tested by controlling the frequency from 0.5 Hz to 20 Hz at 0.8 mA (Figure 4k). Even though the sciatic nerve was stimulated with a comparably fast frequency of 20 Hz using the biodegradable fiber electrode, gastrocnemius muscle was activated accordingly as shown in Video S2 (Supporting Information). Electrical stimulation below 20 Hz can also be used for various applications such as nerve regeneration, sensorimotor function restoration, and neuropathic pain relief.^[^ ^60^ ^—^ ^63^ ^]^ Although the presented biodegradable fiber electrode exhibited the excellent long‐term stability in the PBS solution based on the encapsulating buffer layer (Figure 3g and Figure S20, Supporting Information), future work regarding in‐depth in vivo study of the fiber electrode is further required to demonstrate chronic or semi‐chronic use of the in vivo electrical stimulator using the fiber electrode.

## 3 Conclusion

In summary, we developed a biodegradable fiber electrode based on the surface‐embedding process of Mo microparticles into the PCL scaffold fibers. The biodegradable fiber electrode was fabricated by incorporating a large amount of Mo microparticles into the outermost volume of the PCL scaffold fiber via the surface‐embedding process. The fabricated biodegradable fiber electrode simultaneously exhibited high electrical performance (≈43.5 Ω cm^−1^), robust mechanical strength, and high stability due to the PCL core‐conductive shell structure. We established an analytical model to elaborate the electrical behavior of the biodegradable fiber electrode under the bending deformation. The excellent biocompatibility of the biodegradable fiber electrode was evaluated by the cell viability test using 3T3 fibroblasts cells, and the dissolution property of the fiber electrode was also demonstrated in the PBS test. We successfully demonstrate various practical applications of the fiber electrode as an interconnect, suturable fiber temperature sensor, and fiber‐based in vivo electrical stimulator. Future work on the biodegradable fiber electrode will focus on further improvement of its electrical performance while maintaining the mechanical robustness and stability, to be used for developing high‐performance in vivo electronic devices. Furthermore, various fiber‐based implantable electronic devices, which can be completely dissolved after use without surgical extraction, could be developed using the biodegradable fiber electrode.

## 4 Experimental Section

### Preparation of the PCL Scaffold Fiber

Polycaprolactone (PCL) fiber was fabricated by a melt‐drawing process. Melt drawing process was performed using a custom‐made melt‐drawing system. 40 g of PCL pellets (Signal‐Aldrich Corp., average *M_n_* 80 000, 440 744) were put in a stainless container and melted at 100 °C on a hot plate (MISUNG SCIENTIFIC Co., HS15) for 1 hour. After 1 hour, a glass rod fixed in the custom‐made melt drawing machine was vertically immersed in the melted PCL for 10 s. The melted PCL was drawn in the form of a fiber by pulling a glass rod from the melted PCL liquid at 4 mm s^−1^.

### Cold Drawing Process of the PCL Scaffold Fiber

For cold‐drawing of the drawn PCL scaffold fiber, one end of the drawn PCL fiber was fixed and the other end of the PCL fiber was pulled in the longitudinal direction using a custom‐made drawing machine. The drawing machine pulled the PCL fiber until the length of the PCL fiber was elongated up to 320%. To achieve high uniformity of cold drawing over the fiber, the PCL fiber was locally heated using a heating gun at 50 °C during the cold drawing process. After the cold drawing process, the elongated PCL fiber was cured in a vacuum dry oven (SH SCIENTIFIC Co., SH‐VDO‐216NG) at 60 °C for 1 h.

### Surface‐Embedding Process of Mo Microparticles on the Cold‐Drawn PCL Fiber

For the surface‐embedding of Mo microparticles, the cold‐drawn PCL fiber was immersed in a solution of molybdenum microparticles (10 wt%) (Goodfellow, particle size: maximum 105 µm, purity 99.9+%) and tetraethylene glycol (10 wt%) (Sigma‐Aldrich Corp., 99%, 110 175) in tetrahydrofuran (DAEJUNG, CAS. No. 109‐99‐9) at 35 °C. The solution including the cold‐drawn PCL fiber was vigorously shaken for 5s to embed Mo microparticles into the surface of the cold‐drawn PCL fiber. Then, fiber was taken out and dried for 10 min at room temperature. The Mo microparticles‐embedded PCL fiber was additionally cured in a vacuum dry oven at 60 °C in order to remove residual solvent from the fiber.

### Characterization of the Biodegradable Fiber Electrode

The stress–strain curves of the PCL fibers and fiber electrodes were measured using a custom‐made stretching machine with a Force‐meter (IMADA Inc., DSV‐20N) with a stretching speed of 0.1 mm s^−1^. The electrical resistance of the fiber electrode was measured using an inductor–capacitor–resistor (LCR) meter (Keysight‐Technologies Inc., E4980AL). To perform the bending stability and durability of the fiber electrodes, a custom‐made bending stage was used to induce bending deformation of the fiber electrode and the electrical resistance of the fiber electrode was simultaneously measured using an LCR meter. The surface morphologies of the biodegradable fiber electrodes were investigated using a field emission scanning electron microscope (Hitachi Inc., S‐4800) coupled with EDS module (Hitachi Inc.) to obtain SEM images and the corresponding EDS mapping images. The components of the biodegradable fiber electrode were investigated by X‐ray photoelectron spectroscopy (Thermo Scientific Inc., ESCALAB 250Xi). The weight fraction of Mo microparticles in the conductive composite layer of the fiber electrode was measured using thermogravimetric analysis (Ta Instruments Inc., Auto Q500).

### Finite Element Method Simulation

The numerical simulation that can explain the behavior of volume change of bent fiber electrode in tensile and compressive regions through the strain and stress distribution was carried out based on 3D modeling using a COMSOL Multiphysics (version 6.0, https://www.comsol.com). The fiber electrode is modeled as a core–shell structure. The length of the fiber is set as 55 mm. The PCL fiber and fiber electrodes are assumed as homogeneous and linear elastic material, ignoring any uncertainties in the material such as the irregular pore distribution and stochasticity that might occur in the manufacturing process. The simulation is performed in the linear elastic regime, assuming major nonlinearity involved in the simulation is only originated from the large deformation. The material properties of the cold‐drawn PCL fiber are as follows: density = 1145 kg m^−3^, Poisson's ratio = 0.3, and Young's modulus = 165 MPa. Likewise, the material properties of the conductive composite layer are set as follows: density = 1675.4 kg m^−3^, Poisson's ratio = 0.2822, and Young's modulus = 49.34 MPa. All the values are based on the experimental measurement. The displacement boundary condition is given at each side of the fiber: One side is fixed while the ramp displacement from 0 to 30 mm is given to the other side. Based on the axial strain and stress distributions, the fiber can be divided into two subregions, experiencing the tensile and compressive regions.

### Biocompatibility Test of the Biodegradable Fiber Electrode

The biocompatibility of the biodegradable fiber electrode was evaluated by both CCK‐8 analysis and the LIVE/DEAD Viability Assay. In detail, 3T3 fibroblasts cells were plated into 24‐well plates with a cell density of 2 × 10^4^ per well. The following day, half of the cell medium was discarded, and ≈ 1 cm PCL fiber sections were added to the plates. After incubating for 1,3,5, and 7 days, both the tests were performed. For CCK‐8 assay, 300 µL of the CCK‐reagent mixed with cell media was added to each well followed by three times of DPBS washed. After that, the cells were incubated for 30 min at 37 °C with 5% CO^2^. As for the LIVE/DEAD assay, a mixture contained 2 × 10^−6^ m of calcein AM and 4 × 10^−6^ m of EthD‐1 was used. Fluorescence images were obtained through an inverted fluorescent microscope (Nikon Ti2, Japan).

### Characterization of the Dissolution Property of the Biodegradable Fiber Electrode

The biodegradable fiber electrode was immersed in PBS solution (Sigma‐Aldrich Corp., pH 7.4, 806552) at 37 °C to investigate the dissolution property of the fiber electrode. To evaluate the weight loss of the fiber electrode in PBS over time, the fiber electrode immersed in the PBS solution was regularly taken out and dried for 2 h at 55 °C in a vacuum dry oven. The weight of the dried fiber electrode was measured using an analytical balance (Mettler Toledo Inc., ME204). The accelerated dissolution test of the biodegradable fiber electrode was also carried out in the same manner at 55 °C.

### In Vivo Biocompatibility and Biodegradability Test

All procedures with mice were approved by the Committee of Animal Research at the Korea Advanced Institute of Science and Technology (KA2022‐024). Mice were caged in rooms with 12 h light/dark cycles and fed ad libitum with food and water. In this study, Male C57BL/6J mice weighing between 25 and 30 g were used in all the experiments. Animals were anesthetized using 4% isoflurane (induction) and 2% isoflurane (maintenance) through inhalation. The animals were placed in a prone position for all procedures, and all surgeries were conducted on the left hindlegs. For both biocompatibility and biodegradability tests, all fiber electrode insertion surgery was performed as follows. First, an incision of the skin at the dorsal thigh was carried out by sterile surgical scissors. After that, a vertical incision at the dorsal thigh muscles was conducted until the sciatic nerve embedded in the muscle was visible. Next, either the biodegradable fiber electrode and the cold‐drawn PCL fiber were tied to the sciatic nerve. Lastly, the incision site was stitched using suture (6‐0 Vicryl, Ethicon). For immunohistology study, all mice were transcardially perfused with 10% formalin after 6 days of fiber embedding, the sciatic nerves sections tied with the fibers were extracted, and were immersed in 10% of formalin (HT501128, Sigma Aldrich) solution overnight at 4 °C. Then, the fixed sciatic nerves were sectioned into 40 µm cross‐sectioned slices. The cross‐sectional sciatic nerve slices were washed three times with PBS, and then permeabilized and blocked in a 3 v/v% donkey serum and 0.3% v/v Triton X‐100 in PBS solution at room temperature for one hour. After that, the slices were incubated in primary antibodies solutions [rabbit anti‐S100 for Schwann cell (1:300; ab52642, Abcam); goat anti‐160 kD neurofilament medium (1:300; ab195658, Abcam) and rabbit anti‐Iba1 for activated macrophages (1:500; ab107159, Abcam)] for 16 h. After three washes of PBS, the slices were incubated in secondary antibody solutions [Alexa Fluor 594 donkey antirabbit 594 (1:300; R37119, ThermoFisher), Alexa Fluor 488 donkey antigoat (1:300; ab150129, Abcam) and Alexa Fluo 488 donkey antirabbit (1:250; ab150073, Abcam)] at room temperature for two hours. Finally, the slides sciatic nerve was mounted on glass slides for observation with mounting medium VECTASHIELD which contained DAPI nuclear staining (H‐1000, Vector Laboratories) and photographed with a confocal microscope (Nikon C2, Nikon). To observe the degree of degradation of the fiber electrode in vivo, biodegradable fiber was extracted after 15 days of implantation. The extracted biodegradable fiber electrode was washed in DI water for 10 min at room temperature and dried for 2 h at 55 °C in a vacuum dry oven. The weight of dried fiber electrode was measured by an analytical balance (Mettler Toledo Inc., ME204).

### Demonstration of the Suturable Temperature Sensor

To directly suture the fiber temperature sensor onto the silicone‐based artificial skin (Biozoa Biological Supply Co.), a commercial nylon‐based medical thread (B. Braun Co., DS39/C0932574) was used as a guiding thread for the suturing process of the fiber sensor. In particular, the fiber sensor was sutured on the skin pad following the guiding thread connected to the front‐end of the fiber sensor (Figure S24, Supporting Information). After the suturing of the fiber sensor, the guiding thread was removed by cutting it, leaving only the fiber sensor sutured on the skin pad. The tissue drag force of the fiber temperature sensor for the suturing process was measured using a custom‐made drag force testing machine consisting of an automated linear x‐stage (SCIENCETOWN Inc., SM1‐0810‐3S) and force meter (IMADA Inc., DSV‐20N) (Figure S25a, Supporting Information). In particular, the tissue drag force was measured using the force meter connected with the front‐end of the fiber sensor while the fiber was pulled through the fixed artificial skin (Figure S25b, Supporting Information). The measured force was normalized by unit circumference of the fiber sensor. The electrical resistance of the suturable temperature sensor was measured by an LCR meter (Keysight‐Technologies Inc., E4980AL) while the fiber temperature sensor sutured onto the skin pad was heated on a hot plate. At the same time, the change in the temperature of the sutured fiber electrode and skin pad was measured in real‐time using an IR camera (Teledyne FLIR LLC., ETS320).

### Demonstration of the Wireless Temperature Sensing System

The biodegradable fiber electrode was fixed perpendicularly to the ground, and 10 wt% polypropylene carbonate (PPC) (Sigma Aldrich Corp., *M_n_* ≈50 000 by GPC, 389 021) solution dissolved in acetone was flowed along the vertical fiber electrode. The PPC‐coated fiber electrode was placed on a 3D‐printed mold to form the loop‐type coil. To fix the loop‐type coil structure of the fiber electrode, a 10 wt% polyvinyl alcohol (PVA) (Sigma Aldrich Corp., *M*_w_ 89 000–98 000, 341 584) solution in water was poured on the fiber electrode on the mold. The coiled structure embedded in the PVA film was mechanically separated from the 3D‐printed mold. The *S*_11_ spectra of the wireless temperature system was wirelessly measured by a network analyser (Keysight‐Technologies Inc., E5063A) while the fiber sensor was heated on a hot plate. At the same time, the change in the temperature of the wireless sensing system was measured in real‐time using an IR camera (Teledyne FLIR LLC., ETS320)

### In Vivo Electrical Stimulation Test of Biodegradable Fiber Electrode

All procedures with mice were approved by the Committee of Animal Research at the Korea Advanced Institute of Science and Technology (KA2022‐024). Mice were caged in rooms with 12 h light/dark cycles and fed ad libitum with food and water. In this study, Male C57BL/6J mice weighing between 25 and 30 g were used in all the experiments. Animals were anesthetized using 4% isoflurane (induction) and 2% isoflurane (maintenance) through inhalation. The animals were placed in a prone position for all procedures, and all surgeries were conducted on the left hindlegs. For fiber electrode insertion, an incision of the skin at the dorsal thigh was carried out by sterile surgical scissors. After that, a vertical incision at the dorsal thigh muscles was conducted until the sciatic nerve embedded in the muscle was visible. The biodegradable fiber electrode was coated with PAA for electrical insulation (Figure S28, Supporting Information). To coat the PAA layer on the cylindrical surface of the fiber electrode, the fiber was fixed perpendicularly to the ground. The 10 wt% PAA (Sigma Aldrich Corp., average *M*_v_ ≈450 000, 181 285) solution dissolved in ethanol (DAEJUNG, 99.9%, CAS. No. 64‐17‐5) flowed along the vertical fiber electrode, exposing the tip (≈5 mm) of the fiber for the electrical stimulation. The coated fiber was heated in an oven at 60 °C for 60 min to evaporate residual solvent. The biodegradable fiber electrode was then carefully implanted underneath the exposed section of the sciatic nerve without damaging the nerve bundles. For EMG recording, two electrodes made with stainless steel wires were placed in the Tibialis Anterior (TA) muscles of the left hindlegs. In order to properly place the electrodes into muscles, each electrode was threaded through a sterile hypodermic needle with a hook at the needle tip in order to secure the electrodes inside the muscle. The needle was then inserted into the intended muscle and was removed when the electrode was firmly placed at place. The two electrodes were placed ≈1 mm apart. Electrical stimulation experiments were conducted with an isolated pulse stimulator (A‐M system Model 2100, USA), connected to a biodegradable fiber. EMG recordings were recorded at 750 Hz using a Lab Rat LR‐10 acquisition system (Tucker Davis Technologies, USA). Synchronization of the electrical stimulator output and electrophysiological data was collected using SynapseLite (Tucker Davis Technologies, USA). Video recordings were simultaneously obtained with EMG recordings. Electrophysiological data analysis was performed using customized MATLAB scripts in MATLAB (Mathworks, USA).

### Statistical Analysis

Statistical data in all experiments is presented as mean ± SD (n = 3). Origin Pro 2020b software (OriginLab Corp., USA) was used to perform the statistical analyses for all experiments except for in vivo studies, which were performed using Prism software (GraphPad Software Inc. USA). The significant differences between groups were compared by using a one‐way analysis of variance (ANOVA) (*P*‐value > 0.05).

## Conflict of Interest

The authors declare no conflict of interest.

## Supporting information

Supporting Information

Supplemental Video 1

Supplemental Video 2

## Data Availability

The data that support the findings of this study are available from the corresponding author upon reasonable request.
