# Supplementary material for: Surface‐Embedding of Mo Microparticles for Robust and Conductive Biodegradable Fiber Electrodes: Toward 1D Flexible Transient Electronics
Source: Adv Sci (Weinh). 2023 Mar 30;10(15):2206186. doi: 10.1002/advs.202206186 (PMC10214255; doi:10.1002/advs.202206186)
Supplement: Supplementary file 1 — Supporting Information [file ADVS-10-2206186-s003.pdf]

ADVANCED SCIENCE
Open Access

Supporting Information

for *Adv. Sci.*, DOI 10.1002/advs.202206186

Surface-Embedding of Mo Microparticles for Robust and Conductive Biodegradable Fiber Electrodes: Toward 1D Flexible Transient Electronics

*Jinho Kim, Congqi Yang, Taehyun Yun, Seohyun Woo, Hwajoong Kim, Mugeun Lee, Minji Jeong, Hyeji Ryu, Namjung Kim, Seongjun Park and Jaehong Lee**

Supporting Information

Surface-embedding of Mo microparticles for robust and conductive biodegradable fiber electrodes: Toward 1D flexible transient electronics

*Jinho Kim, Congqi Yang, Taehyun Yun, Seohyun Woo, Hwajoong Kim, Mugeun Lee, Minji Jeong, Hyeji Ryu, Namjung Kim, Seongjun Park, and Jaehong Lee**

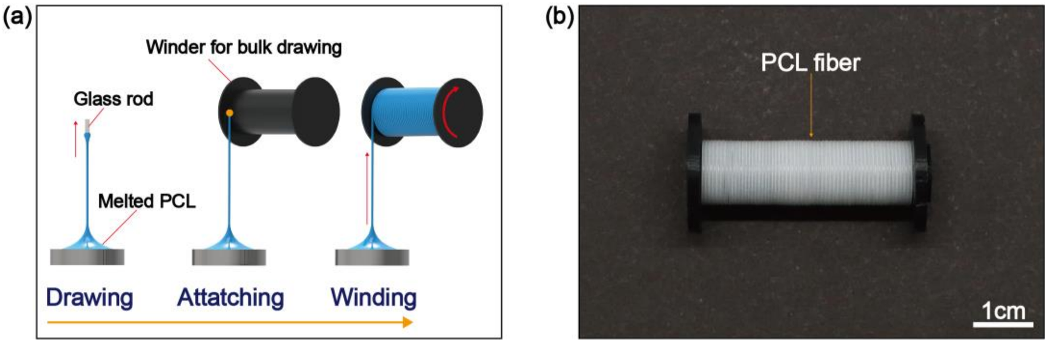

Figure S1. (a) Schematic illustration of fabrication process of the PCL scaffold fiber via the melt drawing process. The melted PCL was extracted by conversion of the rotational motion of the winder to drawing motion. b) Photograph of the fabricated PCL fiber through bulk production.

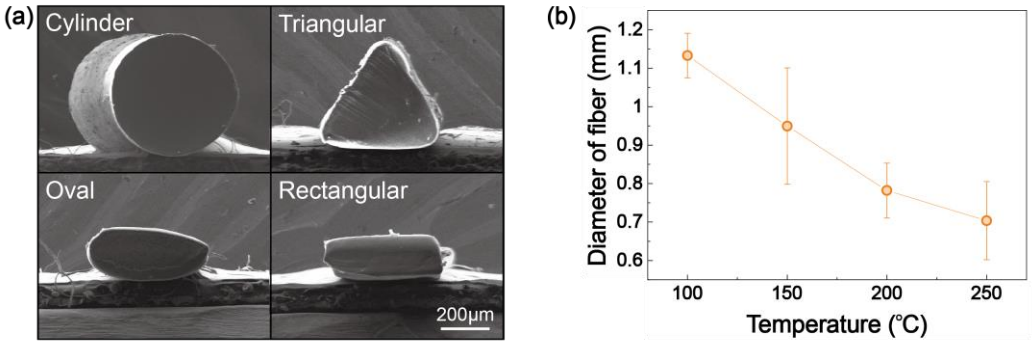

Figure S2. (a) Cross sectional SEM images of the fabricated PCL fiber with various shapes (cylinder, triangular, oval, rectangular structure). (b) Diameter of the drawn PCL fibers according to the increasing melting temperature. Data are presented as mean ± SD (n=3).

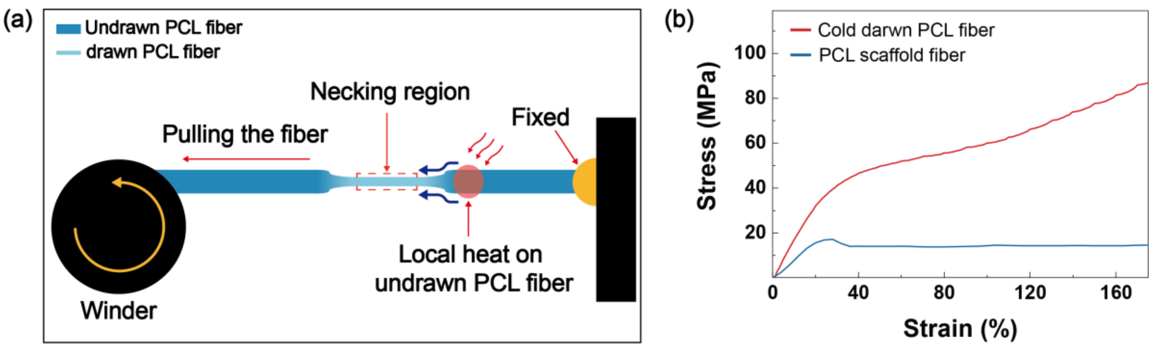

Figure S3. Schematic illustration of the cold drawing process of the PCL scaffold fiber fabricated by the melt drawing process. Tensile stress below the yielding point of PCL was applied to the PCL fiber while the region of undrawn PCL fiber was locally heated at 50 °C. Necking was preferentially initiated from the locally heated region. After the cold drawing process, the locally heated region was cooled down by ambient air, recovering the mechanical properties of the cold-drawn PCL fiber. Thus, the PCL fiber was uniformly elongated over the region of undrawn PCL fiber, increasing the entire length of the PCL fiber to 320% of the original length. (b) Stress-strain curves of cold-drawn PCL fiber (Red line) and PCL scaffold fiber (Blue line).

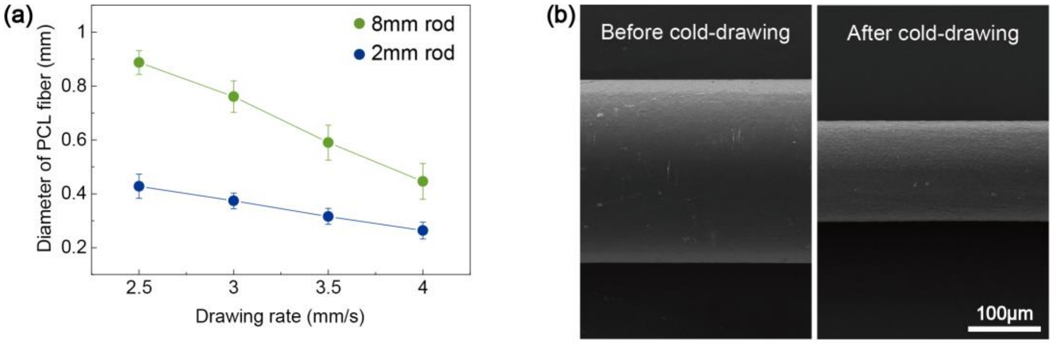

Figure S4. (a) Diameter change of the melt-drawn PCL scaffold fibers fabricated using glass rods with different diameters of 2 and 8 mm with respect to various drawing speeds. Data are presented as mean ± SD (n=3). (b) SEM images showing the PCL scaffold fiber fabricated using a glass rod with 2 mm diameter (left) and the corresponding cold-drawn PCL fiber (right). The diameters of both the PCL scaffold fiber (~260 μm) and the corresponding cold-drawn fibers (~145 μm) were decreased compared with the PCL fibers fabricated using a larger glass rod with 8 mm diameter (Figure 1f).

(a)
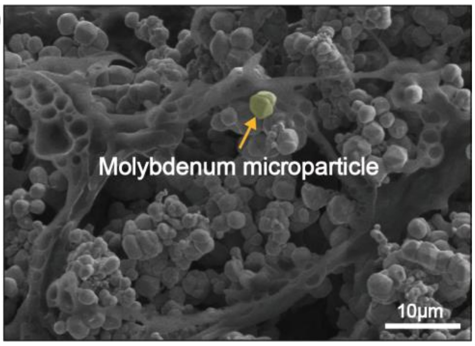

Figure S5. (a) SEM image, showing the surface of the biodegradable fiber electrode. Mo microparticles are uniformly distributed in the conductive composite layer of the biodegradable fiber electrode.

Calculation for electrical conductivity and resistance of the biodegradable fiber electrode based on the percolation theory

Based on the classical percolation theory and power law relationship, the electrical conductivity of the biodegradable fiber electrode can be obtained by calculating the electrical conductivity of the conductive composite between the Mo microparticles and PCL layer in the fiber electrode using the following equation[1]:

$$\sigma = \sigma_0 \left(V_f - V_p\right)^s \qquad \text{(S1)}$$

where σ is the electrical conductivity of the biodegradable fiber electrode, σ_0 is the bulk conductivity of Mo, V_f and V_p are the volume fraction of Mo microparticles in the conductive composite layer and percolation threshold of the biodegradable fiber electrode, respectively, and s (s = 1.8) is a critical exponent. The percolation threshold could be calculated using the following equation as follows[2]:

$$V_p = \frac{1}{(1+4\rho v)} \qquad \text{(S2)}$$

where ρ is the density of the conductive filler (10.28 g cm^{-3}) and v is a specific void space in the random dense-packed bed of filler (0.0621 cm3 g^{-1})[2]. Using Equation S2, the V_p of the biodegradable fiber electrode could be calculated to 0.281. To calculate the volume fraction of Mo microparticles in the conductive composite (V_f), the volume of the conductive composite layer (V_c) and volume of Mo microparticles in the conductive composite layer (V_{Mo}) were calculated as follows:

$$V_c = \pi l (r_{fiber}^2 - r_{core}^2) \qquad \text{(S3)}$$

where l is the length of the biodegradable fiber electrode, and r_{fiber} and r_{core} indicate the radius of the biodegradable fiber electrode and PCL core, respectively. Further, the V_{Mo} could be obtained by calculating the volume of the Mo microparticles in the fiber electrode as follows:

$$V_{Mo} = \frac{m_{fiber} \cdot p_{Mo}}{\rho_{Mo}} \qquad \text{(S4)}$$

where m_{fiber} and p_{Mo} are the weight of the fiber electrode and weight percent of the Mo microparticles in the fiber electrode measured by TGA analysis, respectively, and ρ_{Mo} is the density of the bulk molybdenum (10.28 g cm^{-3}). The weight percent of Mo microparticles was measured at 500 °C, at which PCL and TG in the fiber electrode completely decompose in the TGA measurement. The calculated volume of Mo microparticles in the fiber electrode should be the same with that in the conductive composite layer of the fiber electrode. The calculated volume of Mo microparticles in the fiber electrode should be the same with that in the

conductive composite layer of the fiber electrode. Therefore, the volume fraction of Mo microparticles in the conductive composite layer of the fiber electrode (V_f) can be calculated using the equation (S3) and (S4) as follows:

$$V_f = \frac{V_{Mo}}{V_c} \quad \text{(S5)}$$

By substituting (S2) and (S5) into (S1), the conductivity of the biodegradable fiber electrode can be calculated.

By using the electrical conductivity of the biodegradable fiber electrode calculated by (S1), its electrical resistance can be calculated as follows:

$$R = \frac{1}{\sigma}\frac{l}{A_{cl}} \quad \text{(S6)}$$

where σ is the electrical conductivity of the biodegradable fiber electrode, l is the length of the fiber electrode, and A_{cl} indicates the cross-sectional area of the conductive composite layer in the fiber electrode.

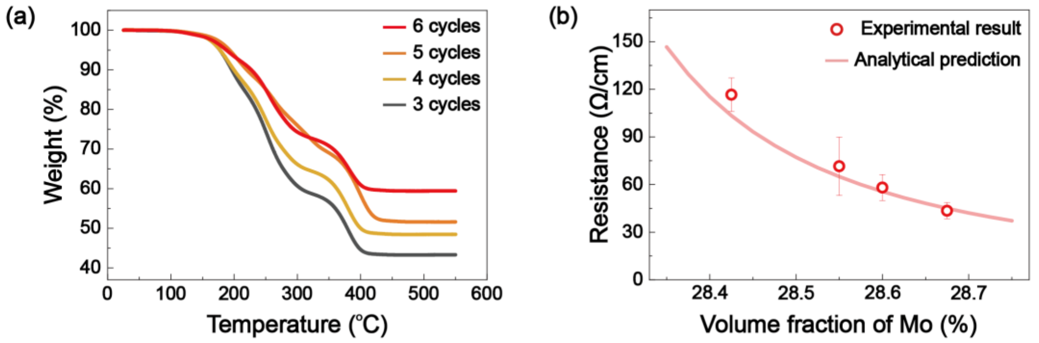

Figure S6. (a) Thermogravimetric analysis (TGA) of the biodegradable fiber electrodes fabricated with different number of repeated surface-embedding cycles. The temperature was increased from 25 °C to 550 °C at a rate of 10 °C min^{-1}. (b) Calculated resistance of the biodegradable fiber electrode by the number of repeated surface-embedding cycles based on the power-law relation and 3D percolation theory. Data are presented as mean ± SD (n=3).

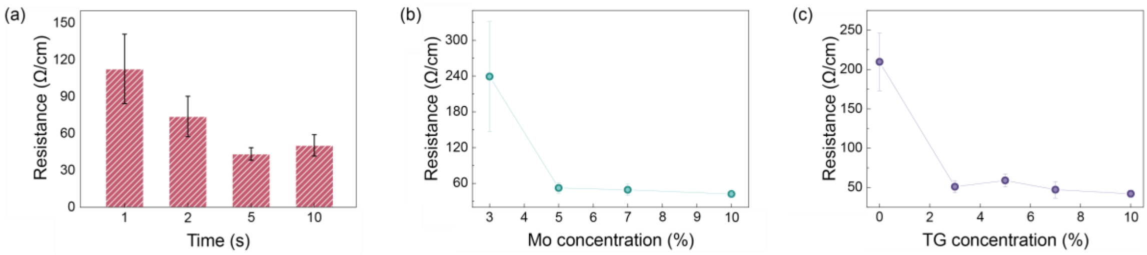

Figure S7. (a) Change in the electrical resistance of the biodegradable fiber electrode according to the reaction time for the surface-embedding process. Data are presented as mean ± SD (n=3). (b) Change in the electrical resistance of the biodegradable fiber electrode with respect to various concentrations of Mo microparticles in the solution used in the surface-embedding process. Data are presented as mean ± SD (n=3). (c) Change in the electrical resistance of the biodegradable fiber electrode according to the concentration of tetraethylene glycol(TG) in the solution used in the surface-embedding process. Data are presented as mean ± SD (n=3).

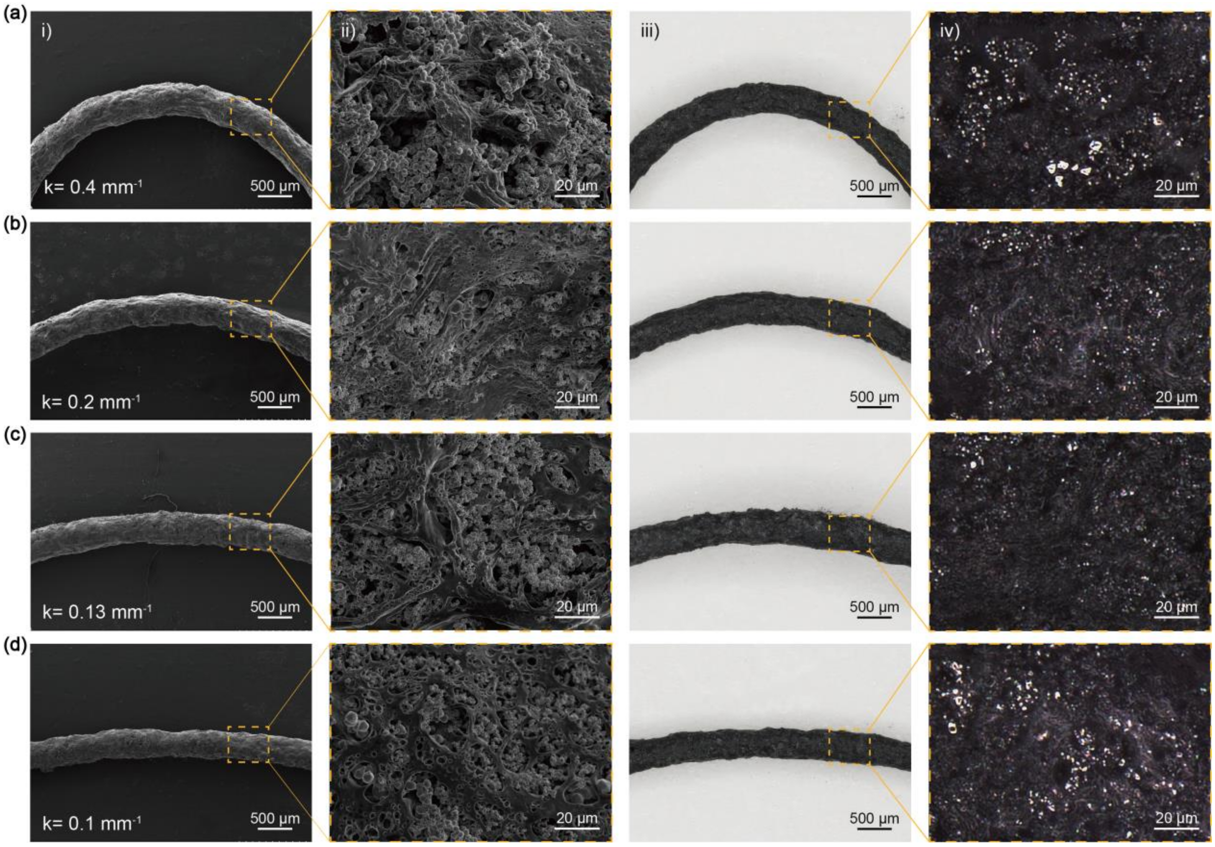

Figure S8. SEM (1, 2 columns) and digital microscopic (3, 4 columns) images showing the surface of the biodegradable fiber electrode according to its increasing curvature from 0.1 to 0.4 mm^{-1}. There is no considerable loss or delamination of the Mo microparticles in the fiber electrode even under various bending deformation, demonstrating high adhesion between the Mo microparticles and PCL matrix.

Analytical solution to calculate the electrical resistance of the biodegradable fiber electrode under bending deformation.

To calculate the electrical resistance of the biodegradable fiber electrode upon bending deformation, the fiber electrode should be considered as two separated regions under the deformation. In particular, when the fiber electrode is bent, the fiber electrode can be divided into tensile and compressive regions according to the applied deformation. Because the distribution of Mo microparticles in each region of the bent fiber is simultaneously changed upon bending deformation, the electrical conductivity of the fiber is locally changed (Figure 2h). Therefore, the electrical circuit for the fiber electrode under bending deformation can be described as two parallel-connected resistors corresponded to the local resistors of the tensile and compressive regions in the bent fiber electrode (Figure 2h).

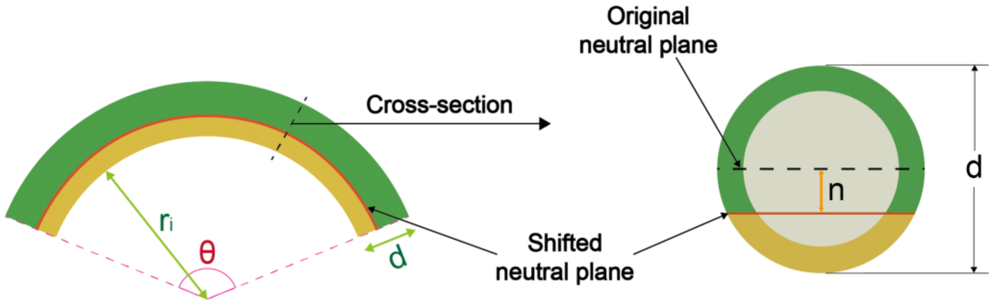

Figure S9. Schematic illustration showing the behavior of the neutral plane upon bending deformation.

To calculate the electrical resistance of the bent fiber electrode analytically, the behavior of the neutral plane in the fiber electrode according to the bending curvature should be considered first. As described in Figure S9, the neutral plane in the bent fiber is shifted towards the concave surface of the bent fiber from the original location. The shifted distance of the neutral plane in the fiber can be derived as follows[3]:

$$n = r_i + \frac{d}{2} - \frac{d^2}{4\left(2\left(r_i+\frac{d}{2}\right)-\sqrt{4\left(r_i+\frac{d}{2}\right)^2-d^2}\right)} \quad \text{(S7)}$$

where the r_i is the bending radius of the bent fiber, and d is the diameter of the biodegradable fiber electrode. Therefore, the volume change of the fiber electrode under bending deformation can be described, as shown in Figure S10a, by converting the bent fiber electrode into a straight form.

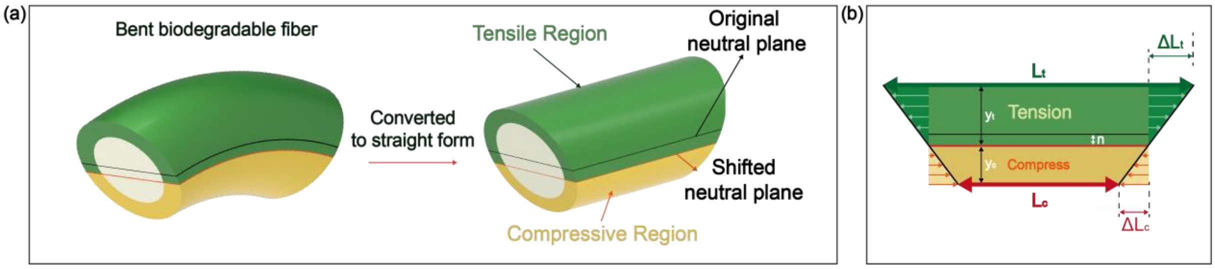

Figure S10. (a) Schematic illustration of bent fiber electrode converted to straight form. (b) Schematic illustration indicating the changed length of the convex and concave surface of the bent fiber electrode.

From Figure S10b, the changed lengths of the convex and concave surface of the bent fiber electrode can be calculated as follows[4]:

$$L_t = L_0 + \Delta L_t = L_0 + L_0\varepsilon_t = L_0(1 + \frac{y_t}{r_i}) = L_0(1 + k\left(\frac{d}{2} + n\right)) \tag{S8}$$

$$L_c = L_0 - \Delta L_c = L_0 - L_0\varepsilon_c = L_0(1 - \frac{y_c}{r_i}) = L_0 - L_0 k\left(\frac{d}{2} - n\right) \tag{S9}$$

where L_t and L_c are the changed lengths of the convex and concave surfaces of the bent fiber, respectively, L_0 is the original length of the fiber electrode on the unbent state, ε_t and ε_c are the maximum tensile and compressive strains in the tensile and compressive regions, respectively, y_t and y_c are the distances from the shifted neutral plane to the convex and concave surfaces of the fiber electrode, respectively, r_i is the bending radius of the bent fiber electrode, and k is the curvature of the bent fiber electrode. Due to the local deformation and shifted neutral plane in the bent fiber electrode, the volumes of the tensile and compressive regions are also changed upon bending deformation. The increased and decreased volumes of the tensile and compressive regions in the bent fiber can be defined as cylindrical wedges, as shown in Figure S11.

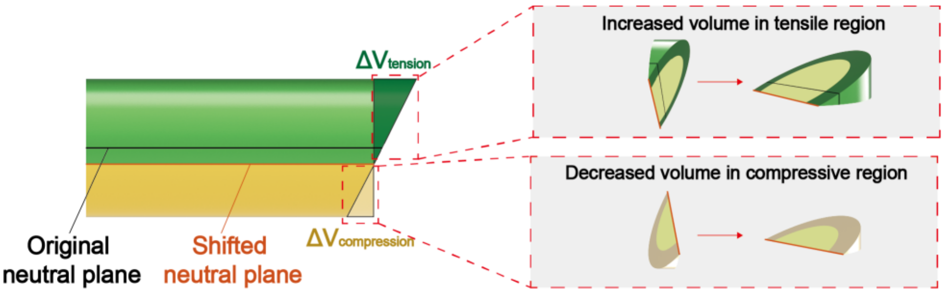

Figure S11. Schematic illustration of changed volumes of the bent fiber electrode in tensile and compressive regions.

To calculate the electrical conductivity of the bent fiber electrodes, the volume change of the conductive composite layer (green and apricot regions in Figure S11) in the bent fiber, which is required for 3D percolation theory, should be calculated. To this end, the volume change of the tensile or compressive region in the bent fiber electrode needs to be calculated by integrating the cross-sectional rectangular area of the truncated cylindrical structure along the x-axis as illustrated in Figure S12a[5,6]:

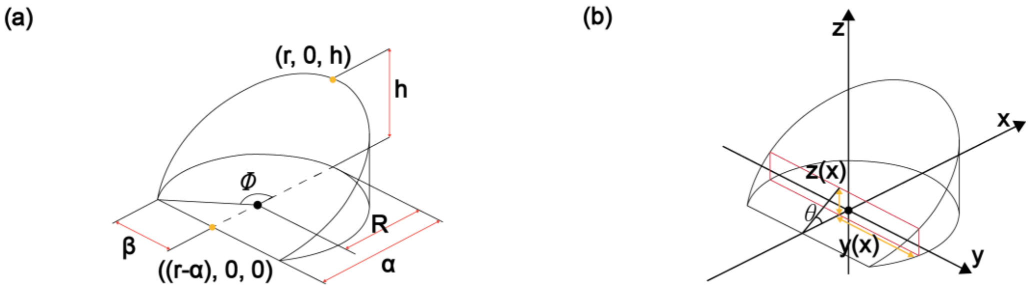

Figure S12. (a) Schematic illustration of the cylindrical wedge explaining the volume change by the bending deformation. (b) Schematic illustration showing the volume integration of the cylindrical wedge.

Therefore, the volume of the truncated cylindrical part (cylindrical wedge), corresponded to the increased volume of the tensile or compressive region in the bent fiber electrode, can be calculated as shown in Figure S12b:

$$\Delta V = \int_{r-\alpha}^{r} z(x) 2y(x) dx \tag{S10}$$

In Equation S10, z(x) can be expressed as the equation for the height in the z-axis of the cross-sectional rectangular area as shown in Figure S13a.

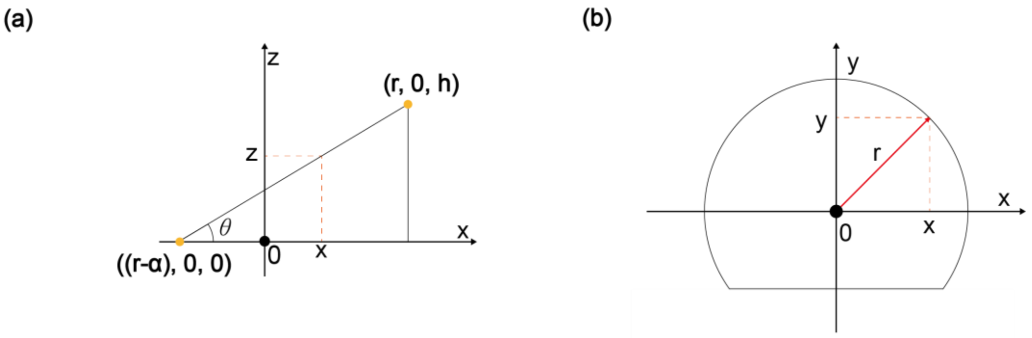

Figure S13. (a) Side view of the cylindrical wedge for calculating the equation of z(x). (b) Top view of the cylindrical wedge evaluating the y(x) using the equation of the circle.

$$z(x) = \tan\theta\left(x - (r-\alpha)\right) = \frac{h}{r-(r-\alpha)}(x-(r-\alpha)) = \frac{h(x-r+\alpha)}{\alpha} \tag{S11}$$

In addition, $y(x)$ can be calculated using the following equation (Figure S13b):

$$y(x) = \sqrt{r^2 - x^2} \tag{S12}$$

By substituting Equation S11 and S12 into Equation S10, the volume of the cylindrical wedge can be calculated as follows:

$$\Delta V = 2\int_{r-\alpha}^{r} \frac{h(x-r+\alpha)}{\alpha} \sqrt{r^2 - x^2}\ dx \tag{S13}$$

The equation S13 can also be simplified as follows[5,6]:

$$\Delta V = \frac{hr^2}{3}\left(\frac{3\sin\emptyset - 3\emptyset\cos\emptyset - \sin^3\emptyset}{1-\cos\emptyset}\right) \tag{S14}$$

where $\emptyset$ indicates the central angle in the projection of the cylindrical wedge on the xy-plane (Figure S13b). The volume changes in the tensile and compressive regions of the bent fiber electrode can be calculated by substituting $h = (\mathrm{r}_{fiber} + \mathrm{n})\tan\theta$ and $h = (\mathrm{r}_{fiber} - \mathrm{n})\tan\theta$ to equation S14.

$$\Delta V_{tf} = \frac{2}{3}(\mathrm{r}_{fiber} + \mathrm{n})r^2\tan\ \theta(\left(\frac{3\sin\emptyset_{tf} - 3\emptyset_{tf}\cos\emptyset_{tf} - \sin^3\emptyset_{tf}}{1-\cos\emptyset_{tf}}\right)) \tag{S15}$$

$$\Delta V_{cf} = \frac{2}{3}(\mathrm{r}_{fiber} - \mathrm{n})r^2\tan\ \theta(\left(\frac{3\sin\emptyset_{cf} - 3\emptyset_{cf}\cos\emptyset_{cf} - \sin^3\emptyset_{cf}}{1-\cos\emptyset_{cf}}\right)) \tag{S16}$$

where, ΔV_{tf} and ΔV_{cf} are the volume change of the tensile and compressive region of the bent fiber electrode, respectively, r is the radius of the biodegradable fiber electrode, n is the shifted distance of the neutral axis, θ indicates the angle of inclination on the cylindrical wedge, and $\emptyset_{tf}$ and $\emptyset_{cf}$ are central angles of the projection of the cylindrical wedges corresponded to the tensile and compressive region in the bent fiber electrode, respectively. By using the equations for the volume changes of the bent fiber electrode, the volume change of the conductive composite layer in the bent fiber electrode can be calculated as follows:

$$\Delta V_{conductive\ layer} = \Delta V_{fiber} - \Delta V_{core} \tag{S17}$$

where, ΔV_{fiber} indicates the equation S15, 16, and ΔV_{core} can also be calculated using the Equation S15 and S16, as follows:

$$\Delta V_{tc} = \frac{2}{3}(\mathrm{r}_{core} + \mathrm{n})\mathrm{r}_{core}^2\tan\ \theta(\left(\frac{3\sin\emptyset_{tc} - 3\emptyset_{tc}\cos\emptyset_{tc} - \sin^3\emptyset_{tc}}{1-\cos\emptyset_{tc}}\right)) \tag{S18}$$

$$\Delta V_{cc} = \frac{2}{3}(\mathrm{r}_{core} - \mathrm{n})\mathrm{r}_{core}^2\tan\ \theta(\left(\frac{3\sin\emptyset_{cc} - 3\emptyset_{cc}\cos\emptyset_{cc} - \sin^3\emptyset_{cc}}{1-\cos\emptyset_{cc}}\right)) \tag{S19}$$

where, ΔV_{tc} and ΔV_{cc} mean the volumes of the cylindrical wedges corresponded to the tensile and compressive region of the PCL core in the bent fiber electrode, respectively, r_{core} is the radius of the PCL core fiber in the bent fiber electrode, and $\emptyset_{tc}$ and $\emptyset_{cc}$ are the central angles of the projection of the tensile and compressive region of PCL core in the bent fiber electrode.

Therefore, the volume change of the conductive composite layer in the tensile and compressive regions of the bent fiber electrode can be calculated as follows:

$$\Delta V_{cc,tensile} = \Delta V_{tf} - \Delta V_{tc} \quad \text{(S20)}$$

$$\Delta V_{cc,compressive} = \Delta V_{tf} - \Delta V_{tc} \quad \text{(S21)}$$

Therefore, the electrical conductivities of the tensile and compressive regions of the bent fiber electrode can be calculated by considering the changed volume of the conductive composite layer in the bent fiber electrode in 3D percolation theory as follows:

$$\sigma_t = \sigma_0 \left(\frac{V^0_{Mo,\ tension}}{V^0_t + \Delta V_{tension}} - V_p \right)^s = \sigma_0 \left(\frac{V_{Mo}}{V_t} - V_p \right)^s \quad \text{(S22)}$$

$$\sigma_c = \sigma_0 \left(\frac{V^0_{Mo,\ compression}}{V^0_c - \Delta V_{compression}} - V_p \right)^s = \sigma_0 \left(\frac{V_{Mo}}{V_c} - V_p \right)^s \quad \text{(S23)}$$

where σ_t and σ_c are the electrical conductivities of the tensile and compressive regions in the bent fiber electrode, respectively, σ_0 is the electrical conductivity of Mo, $V^0_{Mo,\ tension}$ and $V^0_{Mo,\ compression}$ are the volume of Mo microparticles before bending deformation in tensile and compressive regions, V^0_t and V^o_c are the volumes before bending deformation in the tensile and compressive regions, respectively, V_t and V_c indicate the volume of the tensile and compressive region of the bent electrode, respectively, V_p is the percolation threshold, and s is the critical exponent.

By using the calculated electrical conductivity of the bent fiber electrode, the electrical resistance of the bent fiber can be readily calculated as follows:

$$R = \frac{1}{\sigma} \frac{L}{A} \quad \text{(S24)}$$

where σ is the electrical conductivity of the fiber electrode, L is the length of the fiber, and A is the cross-sectional area of the conductive composite layer in the fiber electrode. To further improve the calculated expectation, we considered the bent fiber electrode as a series connection of three different parts of the fiber (bending at sides, unbent, bending in the middle), as described in Figure S14a. Because the bent fiber electrode can be simply described as an equivalent circuit of two resistors in parallel as explained in Figure 2h, the bent fiber electrode can also be described as an equivalent circuit of a series with parallel and single resistors (Figure S14b).

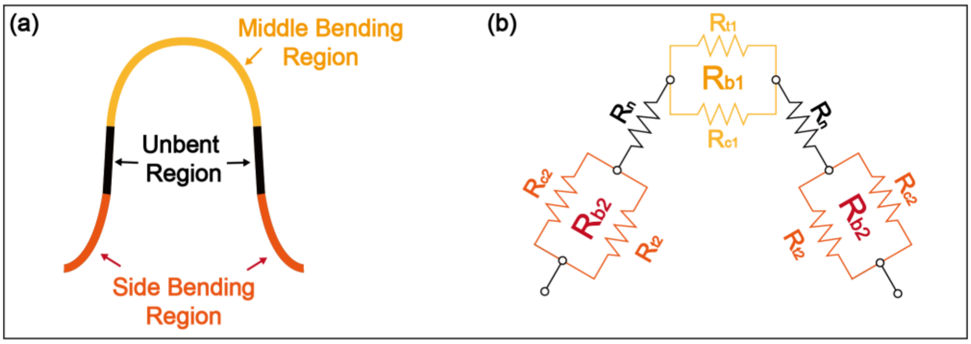

Figure S14. (a) Schematic illustration showing the separation into the middle bending region, unbent region, and side upon the bending deformation. (b) Schematic illustration for the equivalent circuit of the bent fiber electrode regarding middle bending, unbent, and side bending regions.

From Figure S14b, the electrical resistance of the fiber electrode under bending deformation can be calculated as follows:

$$
\begin{aligned}
R_{total} &= R_{b1} + 2R_{b2} + R_n \\
&= \left(\frac{R_{t1}R_{c1}}{R_{t1}+R_{c1}}\right) + 2\left(\frac{R_{t2}R_{c2}}{R_{t2}+R_{c2}}\right) + R_n \\
&= \left(\frac{\left(\frac{1}{\sigma_{t1}}\frac{L_{b1}}{A_{t1}}\right)\left(\frac{1}{\sigma_{c1}}\frac{L_{b1}}{A_{c1}}\right)}{\frac{1}{\sigma_{t1}}\frac{L_{b1}}{A_{t1}}+\frac{1}{\sigma_{c1}}\frac{L_{b1}}{A_{c1}}}\right) + 2\left(\frac{\left(\frac{1}{\sigma_{t2}}\frac{L_{b2}}{A_{t2}}\right)\left(\frac{1}{\sigma_{c2}}\frac{L_{b2}}{A_{c2}}\right)}{\frac{1}{\sigma_{t2}}\frac{L_{b2}}{A_{t2}}+\frac{1}{\sigma_{c2}}\frac{L_{b2}}{A_{c2}}}\right) + \frac{1}{\sigma_n}\frac{L_n}{A_n} \\
&= \left(\frac{L_{b1}}{\sigma_{c1}A_{c1}+\sigma_{t1}A_{t1}}\right) + \left(\frac{2L_{b2}}{\sigma_{c2}A_{c2}+\sigma_{t2}A_{t2}}\right) + \frac{1}{\sigma_n}\frac{L_n}{A_n} \\
&= \frac{1}{\sigma_0}\left[\frac{L_{b1}}{\left(\frac{V^0_{Mt1}}{V_{t1}}-V_p\right)^s A_{t1}+\left(\frac{V^0_{Mc1}}{V_{c1}}-V_p\right)^s A_{c1}} + \frac{2L_{b2}}{\left(\frac{V^0_{Mt2}}{V_{t2}}-V_p\right)^s A_{t2}+\left(\frac{V^0_{Mc2}}{V_{c2}}-V_p\right)^s A_{c2}} + \frac{L_n}{\left(V_f-V_p\right)^s A_n}\right] \quad \text{(S25)}
\end{aligned}
$$

where R_{b1} and R_{b2} are the electrical resistance of the bent part in the middle and sides, respectively, R_n indicates the electrical resistance of the unbent fiber part of the bent fiber electrode, L_{b1} and L_{b2} are length of the bent part in middle and sides, repectively, L_n is a length of the unbent part, V^0_{Mc1} and V^0_{Mc2} are are the volume of Mo microparticles before bending deformation in compressive region regarding middle and sides, respectively, and A_{t1} and A_{t2} indicate the area of bent part in tensile region regarding middle and sides, A_{c1} and A_{c2} indicate the area of bent part in compressive region regarding middle and sides, respectively, and A_n is the area of unbent fiber. The slight deviation between the experimental data and analytical prediction can be because our analytical model considered the 2-dimensional bending motion of the fiber electrode.

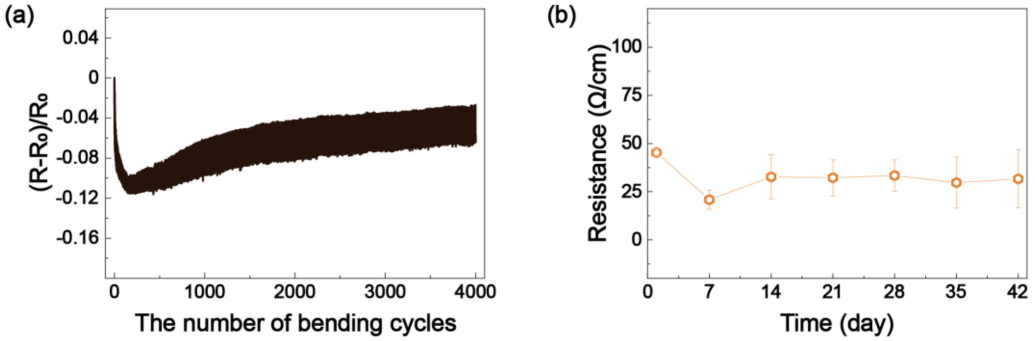

Figure S15. (a) Durability test of the resistive response of the biodegradable fiber electrode during 4,000 bending cycles from 0.04 mm^{-1} of curvature to 0.09 mm^{-1} of curvature. (b) Long-term stability of the biodegradable fiber electrode with respect to electrical resistance in air condition. Data are presented as mean ± SD (n=3).

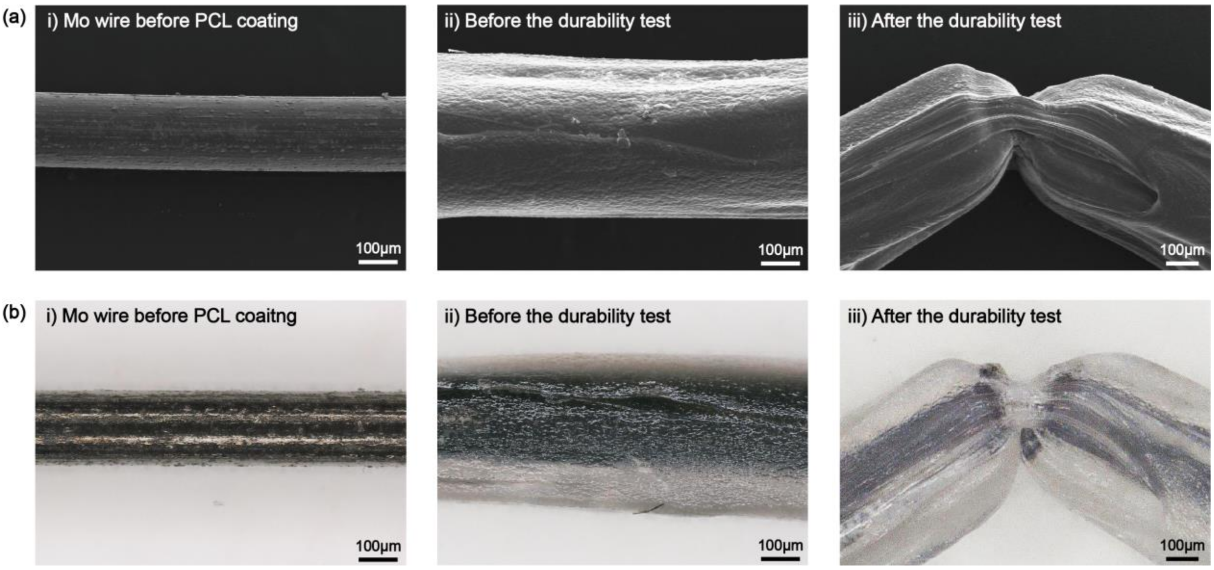

Figure S16. (a) SEM and (b) digital microscopic images showing a PCL-coated Mo wire i) before and ii) after a ~1,200 bending cyclic test with a curvature of 0.3 mm^{-1}. For the cyclic test, a thin Mo wire (09one Inc., Molybdenum wire) with diameter of 200 µm was coated with PCL layer with thickness of 50 µm. After the repeated bending cycles, the PCL-coated Mo wire was mechanically ruptured, showing poor durability of the Mo wire.

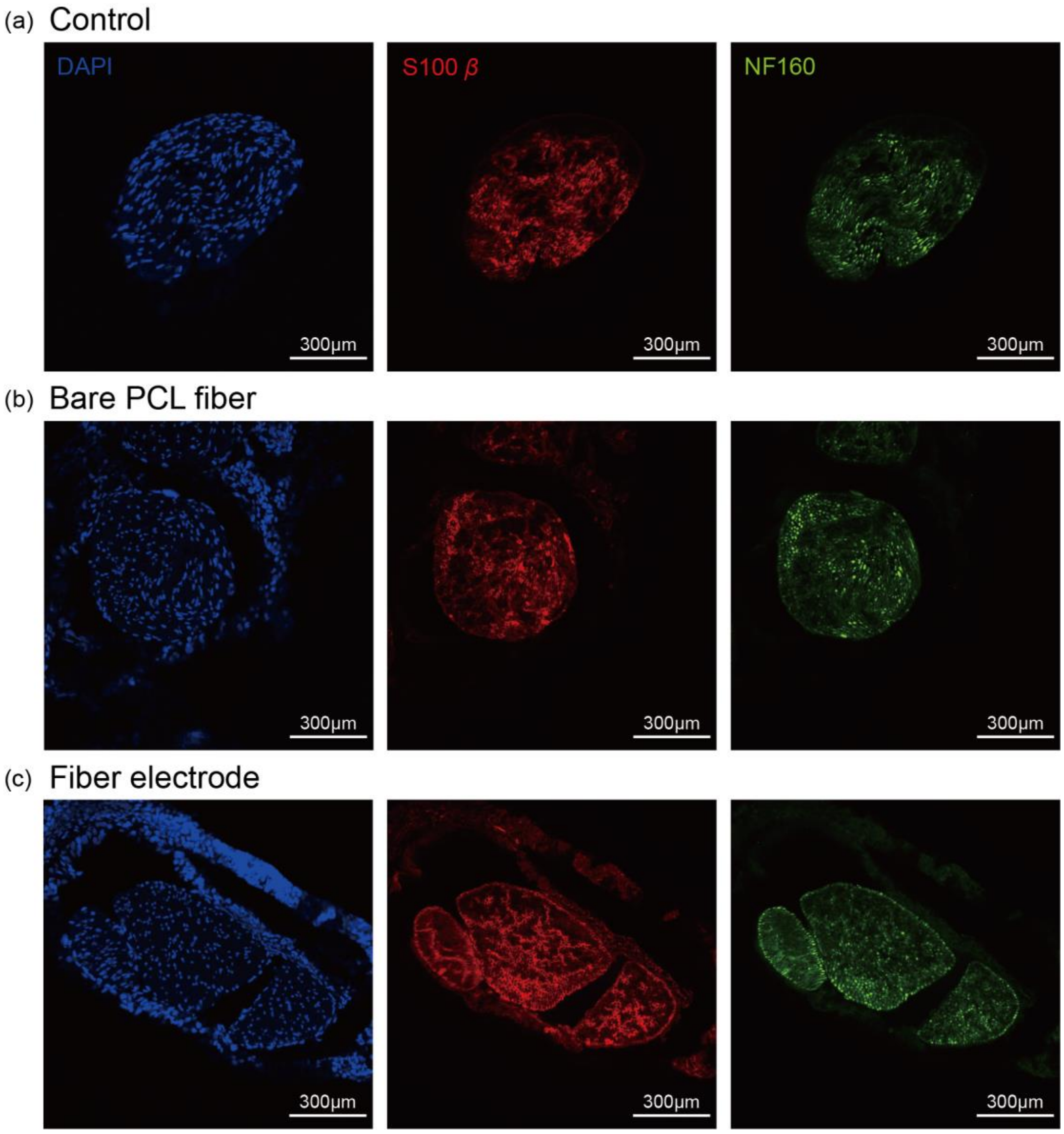

Figure S17. Representative images of NFM(Neurofilament) and S100(Schwann cell) double immunostaining of sciatic nerves after 6 days of implantation for (a) control, (b) bare PCL fiber, and (c) fiber electrode. Blue, DAPI. Red, S100. Green, NFM.

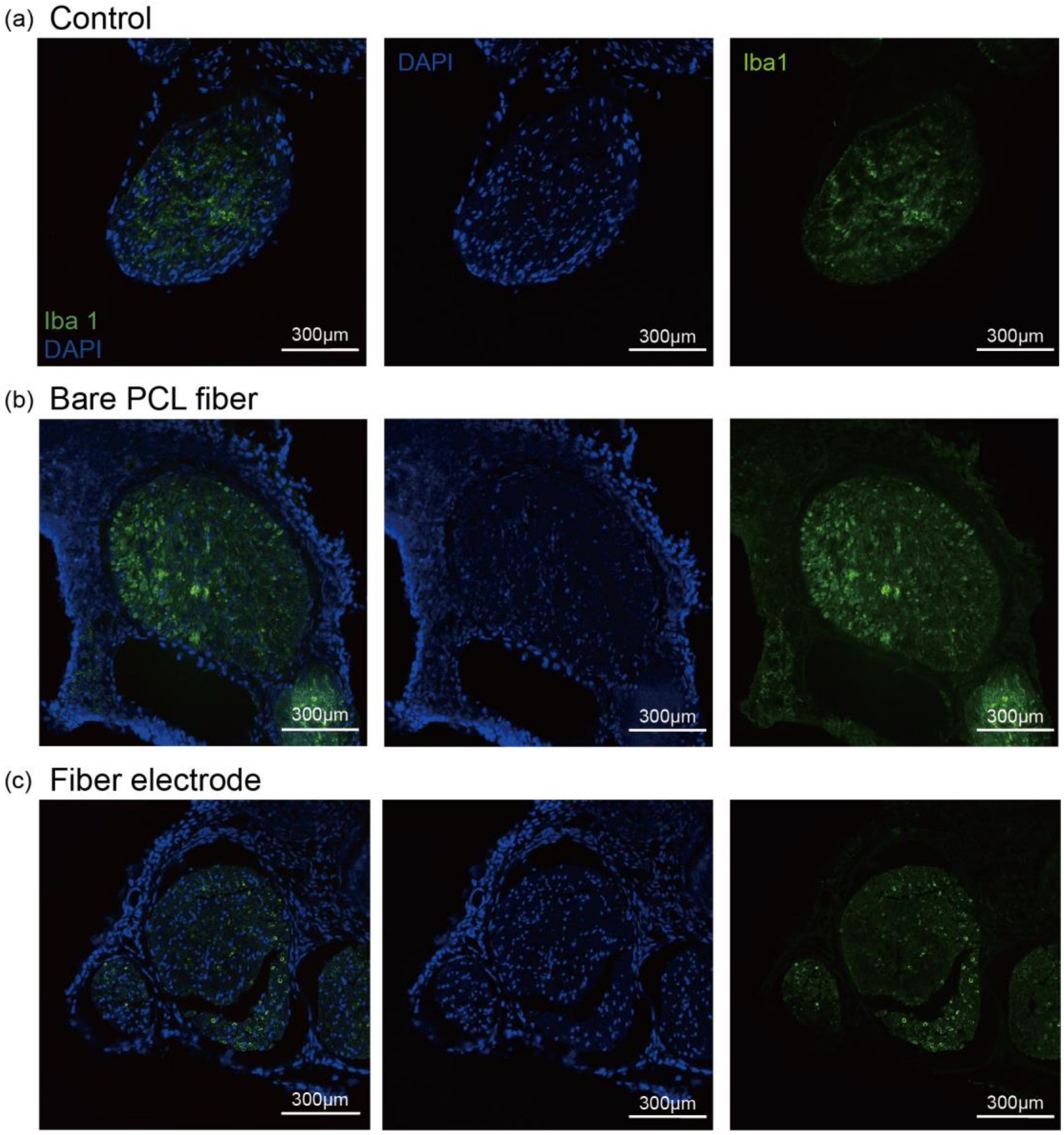

Figure S18. Representative images of sciatic nerve immunolabeled for Iba1(macrophage marker) after 6-day of implantation for (a) control, (b) bare PCL fiber, and (c) the fiber electrode. Blue, DAPI. Green, Iba1.

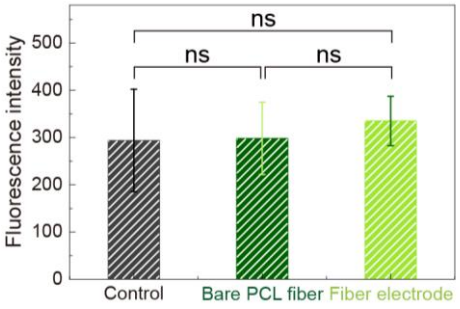

Figure S19. Quantification of the mean fluorescence intensity of cross-section of sciatic nerves that are stained with Iba1 from mice in control, bare PCL fiber and fiber electrode group. Data are presented as mean ± SD (n=3), and statistical analysis proceeded with one-way ANOVA ($P > 0.05$; ns: no significant difference).

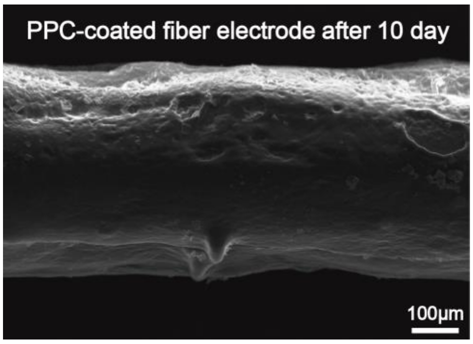

Figure S20. SEM image showing the surface of the PPC-coated fiber electrode after 10 days in the PBS solution. The PPC buffer layer was not fully dissolved after the 10 days, which extended the life time of the fiber electrode in the PBS solution.

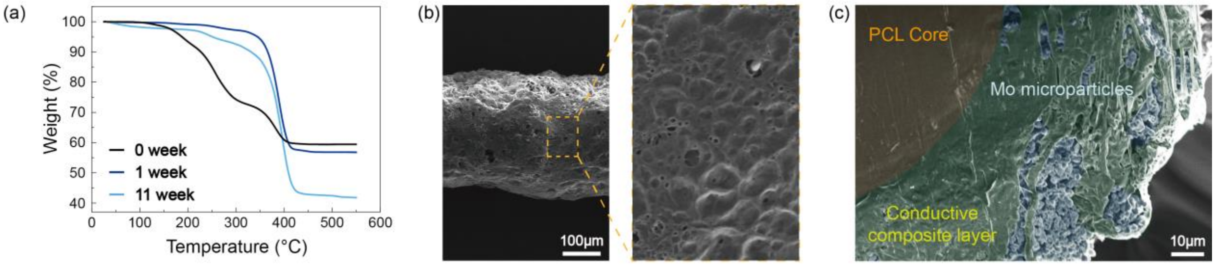

Figure S21. (a) Thermogravimetric analysis (TGA) of the fiber electrode immersed in the PBS solution for 0, 1, and 11 weeks. The fiber electrode contained the Mo microparticles even after 11 weeks of immersion in the PBS solution. (b) Top-view and (c) cross-sectional SEM images of the fiber electrode immersed in the PBS solution for 100 days. The surface of the fiber electrode was covered by the swollen PCL layer, which results in the remaining Mo microparticles in the conductive composite layer even after the immersion in the PBS solution.

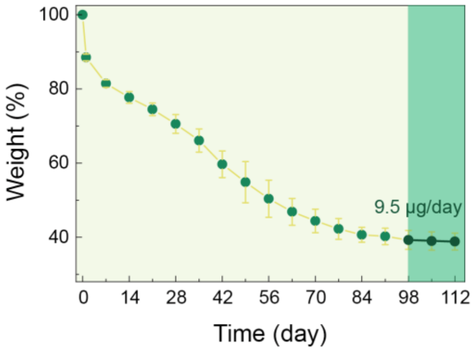

Figure S22. Weight loss of the biodegradable fiber electrode over time in the PBS solution at 37ºC. The weight of the fiber electrode was rapidly decreased until ~98 days mainly due to the dissolution of the conductive composite layer in the fiber. After 98 days, the dissolution rate of the fiber electrode was decreased and saturated to ~9.5 μg/day, corresponded to the dissolution of the PCL core. Data are presented as mean ± SD (n=3).

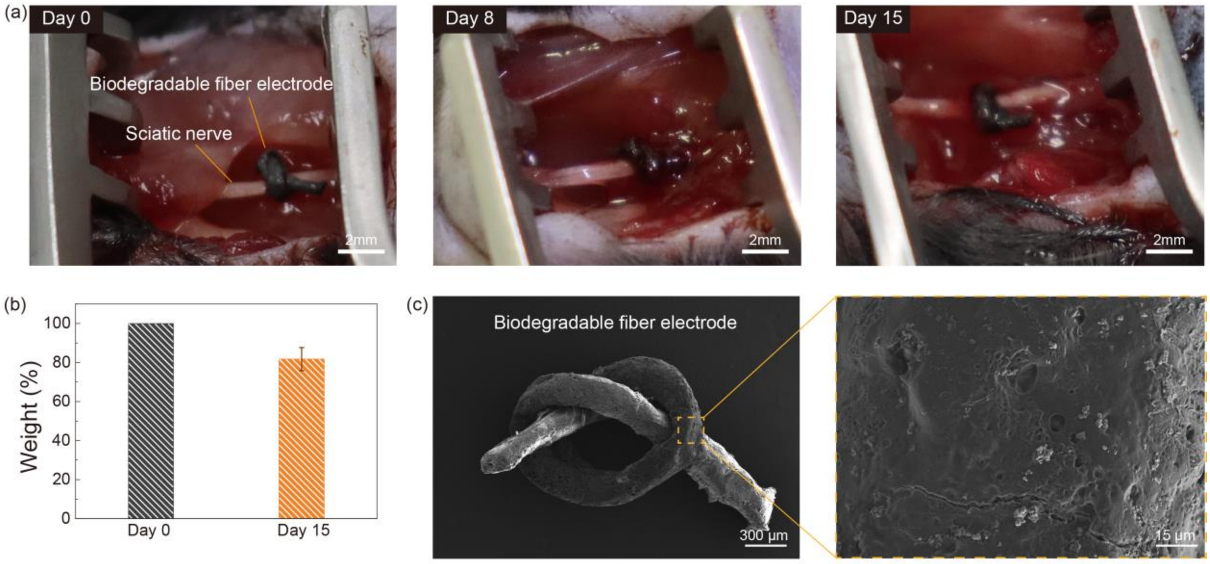

Figure S23. (a) Photographs of the in-vivo biodegradation of the fiber electrode on the sciatic nerve of a rat at 0, 8, and 15 days after the implantation. (b) Weight loss of the biodegradable fiber electrode implanted on the sciatic nerve after 0 and 15 days of the implantation. Data are presented as mean ± SD (n=3). (c) SEM images of the fiber electrode implanted on the sciatic nerve after 15 days of the implantation.

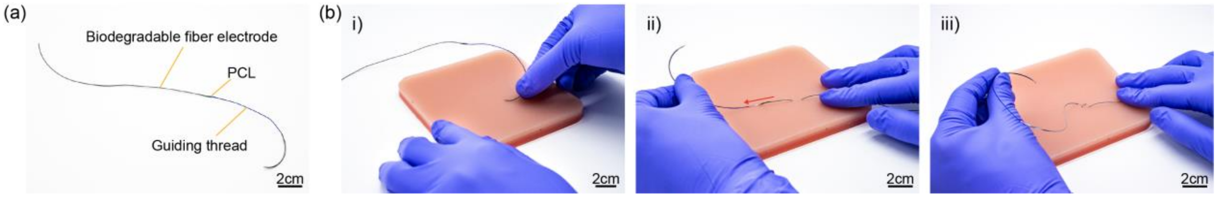

Figure. S24. (a) Photograph of the biodegradable fiber electrode connected with a nylon-based medical thread as a guiding thread using a PCL-based adhesive. (b) Photographs showing the suturing process of the biodegradable fiber electrode on an artificial skin pad using the guiding thread in the suturing system.

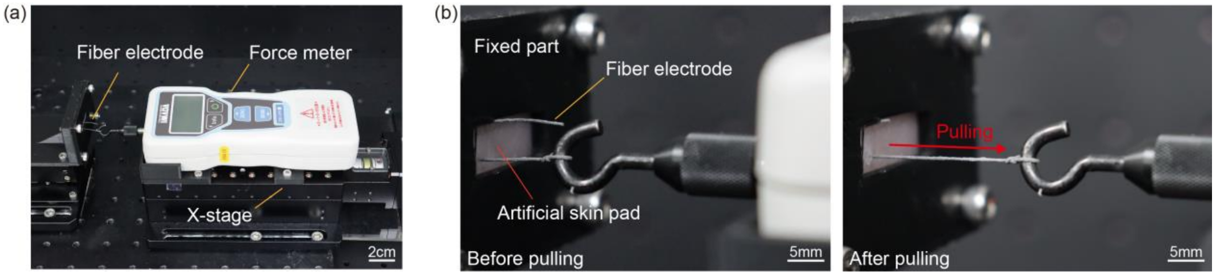

Figure S25. (a) Photograph showing the custom-made drag force measurement system consisting of a linear x-stage and force meter. (b) Photographs showing the measurement of the tissue drag force of the fiber electrode. The tissue drag force was measured by the force meter connected to the fiber electrode while the fiber is slowly pulled through the artificial skin pad attached on a fixed part in the measurement system.

Calculation of change in the electrical resistance of the biodegradable fiber electrode under varying temperature

The change in the electrical resistance of the biodegradable fiber electrode at different temperatures can be calculated by considering the volume change of the conductive composite layer in the biodegradable fiber electrode by thermal expansion. To calculate the volume change of the conductive composite layer in the fiber electrode under high temperature, the length change of the fiber electrode by thermal expansion should first be calculated using the following equation[7]:

$$L_{expansion} = L_0(1 + \alpha(T_1 - T_0)) \quad \text{(S26)}$$

where $L_{expansion}$ is the increased length of the biodegradable fiber electrode by thermal expansion, L_0 indicates the initial length of the fiber electrode, α is the coefficient of linear thermal expansion of PCL ($\alpha = 18 \times 10^{-5}\text{°C}^{-1}$)[8], and T_0 and T_1 are the initial and increased temperatures, respectively. Based on Equation S26, the expanded volume of the conductive composite layer in the fiber electrode at increased temperature T_1 can be calculated as follows:

$$\begin{aligned} V_{expanded,cc} &= \pi L\left(r_{fe}^2 - r_{ce}^2\right) \\ &= \pi L_0(1 + \alpha(T_1 - T_0))\left(r_{f0}^2 - r_{c0}^2\right)(1 + \alpha(T_1 - T_0))^2 \\ &= \pi L_0(r_{f0}^2 - r_{c0}^2)(1 + \alpha(T_1 - T_0))^3 \end{aligned} \quad \text{(S27)}$$

where, r_{fe} and r_{ce} are the increased radius of the biodegradable fiber electrode and PCL core fiber, respectively, and r_{f0} and r_{c0} indicate the initial radius of the biodegradable fiber electrode and PCL core fiber, respectively. By substituting Equation S27 into Equation S1 (power law relationship), the electrical conductivity of the fiber electrode at increased temperature T_1 can be calculated as follows:

$$\sigma_{highT} = \sigma_0\left(V_f - V_p\right)^s = \sigma_0\left(\frac{V_{Mo}}{V_{expanded,cc}} - V_p\right)^s \quad \text{(S28)}$$

where, σ is the electrical conductivity of the biodegradable fiber electrode, σ_0 is the electrical conductivity of Mo, V_f and V_p indicate the volume fraction of Mo microparticles in the conductive composite layer and percolation threshold of the biodegradable fiber electrode, respectively, and s (s = 1.8) is a critical exponent. Therefore, the electrical resistance of the fiber electrode at increased temperature T_1 can be calculated as follows:

$$R_{highT} = \frac{1}{\sigma}\frac{L}{A} = \frac{1}{\sigma_0\left(\frac{V_{Mo}}{V_{expanded,cc}} - V_p\right)^s}\frac{L_0(1+\alpha(T_1-T_0))}{A_0(1+\alpha(T_1-T_0))^2} = \frac{L_0}{\sigma_0 A_0(1+\alpha(T_1-T_0))\left(\frac{V_{Mo}}{V_{expanded,cc}} - V_p\right)^s} \quad \text{(S29)}$$

where, A and A_0 are the increased cross-sectional area and initial cross-sectional area of the conductive composite layer in the fiber electrode, respectively.

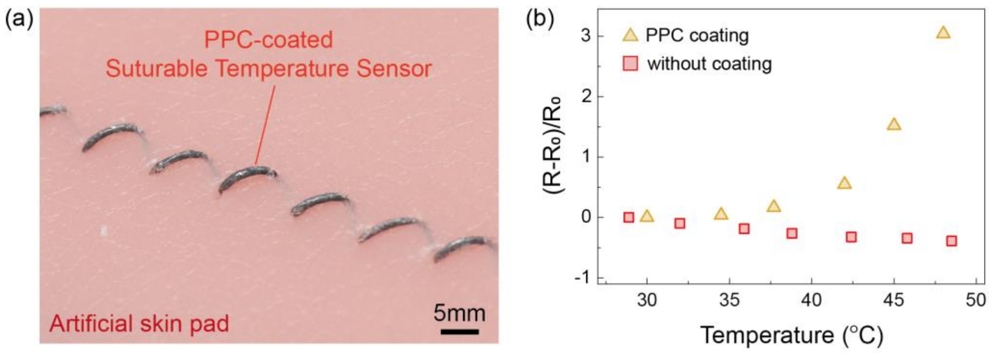

Figure S26. (a) Photograph of the PPC-coated fiber temperature sensor directly sutured onto the artificial skin. (b) Resistive responses of the PPC-coated (Yellow triangle) and uncoated (Red box) fiber temperature sensor sutured on the artificial skin pad by increasing temperature in the PBS solution.

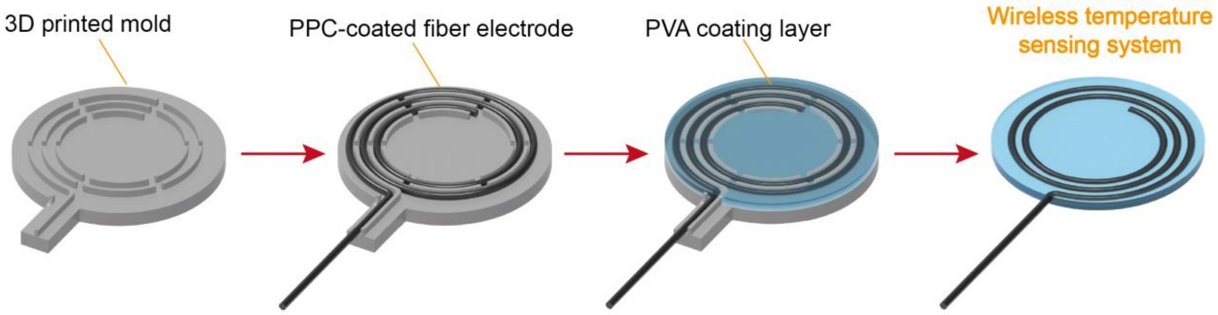

Figure S27. Schematic illustration of a fabrication process of the wireless temperature sensing system using the biodegradable fiber electrode.

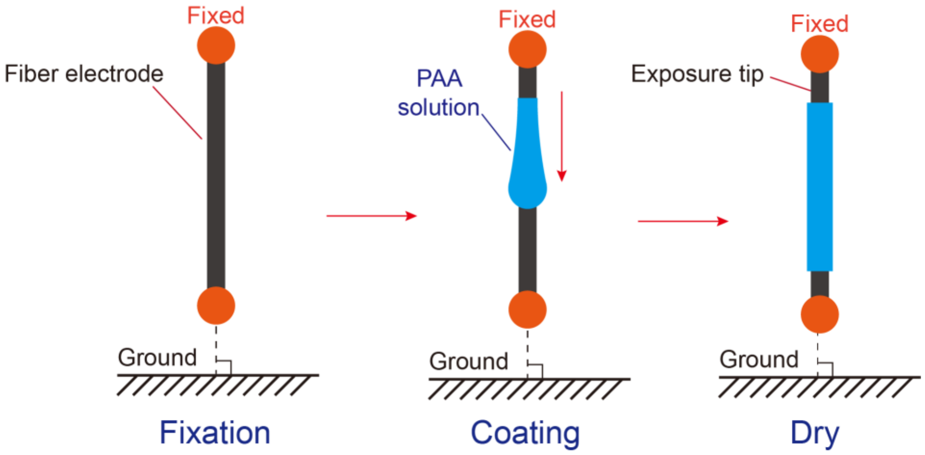

Figure S28. Schematic illustration showing the coating process of PAA solution on the cylindrical surface of the fiber electrode.

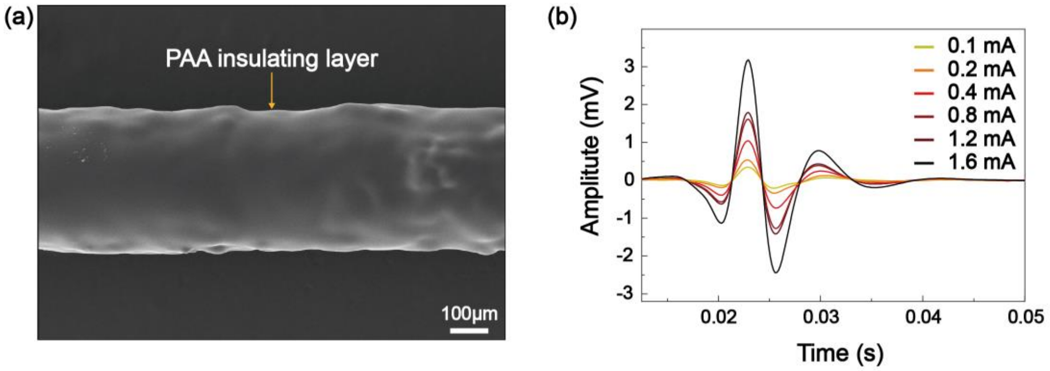

Figure S29. (a) SEM image of the biodegradable fiber electrode coated with poly acrylic acid for insulation. (b) Amplitude change of activated EMG signals by increasing the amplitude of the electrical current at 1 Hz.

References

[1] E. P. Mamunya, V. V. Davidenko, E. V. Lebedev, *Polym. Compos.* **1995**, *16*, 319.

[2] J. Janzen, *J. Appl. Phys.* **1975**, *46*, 966.

[3] A. C. Ugural, S. K. Fenster, *Advanced mechanics of materials and applied elasticity*, Pearson Education, **2011**.

[4] J. M. Gere, B. J. Goodno, *Mechanics of materials*, Cengage learning, **2012**.

[5] W. F. Kern, J. R. Bland, *Solid mensuration: with proofs*, J. Wiley & Sons, Incorporated, **1938**.

[6] J. W. Harris, H. Stöcker, *Handbook of mathematics and computational science*, Springer Science & Business Media, **1998**.

[7] T. Park, H. K. Woo, B. K. Jung, B. Park, J. Bang, W. Kim, S. Jeon, J. Ahn, Y. Lee, Y. M. Lee, T. Il Kim, S. J. Oh, *ACS Nano* **2021**, *15*, 8120.

[8] Q. Ge, X. Luo, C. B. Iversen, H. B. Nejad, P. T. Mather, M. L. Dunn, H. Jerry Qi, *Int. J. Solids Struct.* **2014**, *51*, 2777.
